# Photocatalytic Air Purification Using Functional Polymeric Carbon Nitrides

**DOI:** 10.1002/advs.202102376

**Published:** 2021-10-24

**Authors:** Min Zhou, Honghui Ou, Shanrong Li, Xing Qin, Yuanxing Fang, Shun‐cheng Lee, Xinchen Wang, Wingkei Ho

**Affiliations:** ^1^ Department of Science and Environmental Studies The Education University of Hong Kong Tai Po, New Territories Hong Kong P. R. China; ^2^ Department of Chemistry Tsinghua University Beijing 100084 P. R. China; ^3^ State Key Laboratory of Photocatalysis on Energy and Environment College of Chemistry Fuzhou University Fuzhou 350116 P. R. China; ^4^ Department of Civil and Environmental Engineering The Hong Kong Polytechnic University Hong Kong P. R. China

**Keywords:** denitrification, desulfurization, photocatalytic air purification, polymeric carbon nitrides, volatile organic compound removal

## Abstract

The techniques for the production of the environment have received attention because of the increasing air pollution, which results in a negative impact on the living environment of mankind. Over the decades, burgeoning interest in polymeric carbon nitride (PCN) based photocatalysts for heterogeneous catalysis of air pollutants has been witnessed, which is improved by harvesting visible light, layered/defective structures, functional groups, suitable/adjustable band positions, and existing Lewis basic sites. PCN‐based photocatalytic air purification can reduce the negative impacts of the emission of air pollutants and convert the undesirable and harmful materials into value‐added or nontoxic, or low‐toxic chemicals. However, based on previous reports, the systematic summary and analysis of PCN‐based photocatalysts in the catalytic elimination of air pollutants have not been reported. The research progress of functional PCN‐based composite materials as photocatalysts for the removal of air pollutants is reviewed here. The working mechanisms of each enhancement modification are elucidated and discussed on structures (nanostructure, molecular structue, and composite) regarding their effects on light‐absorption/utilization, reactant adsorption, intermediate/product desorption, charge kinetics, and reactive oxygen species production. Perspectives related to further challenges and directions as well as design strategies of PCN‐based photocatalysts in the heterogeneous catalysis of air pollutants are also provided.

## Graphical abstract

Polymeric carbon nitride‐based photocatalysts have shown great potential in indoor air quality remediation. The state‐of‐the‐art developments in structure regulation including electronic structure, nanostructure, and heterostructure, and their effect on the abatement of air pollution, revealing the structure‐performance relationship in environmental catalysis are discussed here.

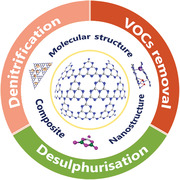

## 1 Introduction

With the increasing recognition of environmental issues, robust attention of the public has been paid to reduce air pollutants.^[^ ^1^, ^2^, ^3^, ^4^, ^5^ ^]^ The undesirable and harmful materials of air pollutants (e.g., nitrogen oxides (NO*_x_*), sulfur compounds, volatile organic compounds (VOC), and other species (carbon monoxide) enter the atmosphere from sources currently beyond human control.^[^ ^6^, ^7^, ^8^, ^9^, ^10^, ^11^, ^12^, ^13^, ^14^, ^15^, ^16^, ^17^, ^18^, ^19^, ^20^ ^]^ These man‐made air pollutants are primarily produced from counts of life and industry (e.g., transportation, fossil fuel combustion, vehicle exhaust, industrially processes, and miscellaneous).^[^ ^2^, ^21^, ^22^, ^23^ ^]^ The quantities effects generated from the harmful air pollutants may seriously damage the global environment, which may induce acid rain, thereby destructing the ozone layer by chlorofluorocarbons; and deteriorate the human/animal health, thereby causing respiratory problems; and increase the form of brown or hazy air or unpleasant smells.^[^ ^24^, ^25^, ^26^, ^27^, ^28^ ^]^ Recently, the control technologies (primarily related to the resources), including exhaust release treatment (e.g., advanced physical adsorption and catalytic particulate biofiltration), and exhaust after treatment (e.g., thermal catalysis), are used to achieve the emission reduction of air pollution.^[^ ^29^, ^30^, ^31^, ^32^, ^33^, ^34^ ^]^ These technologies allow us to increase the population and level of economic activity per person while decreasing the most measured air pollutants concentrations. Nevertheless, the sources of emissions control still have not dealt with the actual problem fundamentally because of the presence and production of air pollution.^[^ ^29^, ^31^, ^35^ ^]^ Developing an economic and environmental technology to address the fundamental problems on the air pollutants is necessary, particularly at low pollutant concentrations of part per billion (ppb) levels typically for indoor and outdoor air concentration. Therefore, recently, the fields of surface/interface chemistry regulations on the multiphase catalysis of air pollutants have advanced significantly to improve the quality of human life and economy, and scientific understanding of the effects and such pollutants is incomplete and is paid less attention to.^[^ ^36^, ^37^, ^38^, ^39^ ^]^

**Figure 1:**
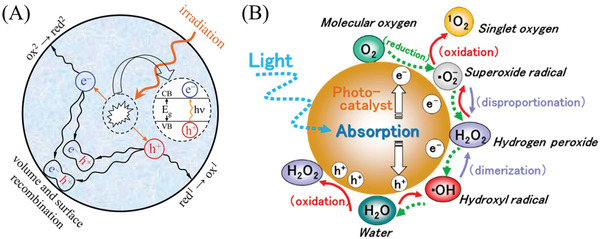
A) The mechanism of the semiconductor‐photocatalyzed reaction. Reproduced with permission.^[^ ^53^ ^]^ Copyright 2007, Royal Society of Chemistry. B) Reactive oxygen species are generated in the photoredox steps. Reproduced with permission.^[^ ^67^ ^]^ Copyright 2017, American Chemical Society.

To date, numerous studies have reported that heterogeneous catalytic treatments used for atmospheric environmental issues are identified as the most efficient solution because of their high efficiency and selectivity.^[^ ^40^, ^41^, ^42^, ^43^, ^44^ ^]^ Among potential and green solutions, semiconductor‐based photocatalysis, which is widely used in atmospheric environment protection, has emerged with inestimable superiority because of its economic, renewable, clean, and safe properties.^[^ ^29^, ^45^, ^46^, ^47^, ^48^, ^49^, ^50^, ^51^ ^]^ Given the fundamental mechanism of multiphase photocatalysis removal of air pollutants (**Figure** **1**A), the process under irradiation can be simplified and divided into three steps: 1) light absorption (fs level, followed by generating the electron–hole couples), 2) reagents’ adsorption (ps–ns level, followed by separating the electron–hole couples), and 3) surface/interface redox reaction (ms–*μ*s level, followed by desorption of the products).^[^ ^52^, ^53^, ^54^, ^55^, ^56^, ^57^ ^]^ In brief, factors such as the optical absorption performance of the semiconductor, the generation/separation/migration of light‐excited carriers, and the reactants adsorption and products desorption seriously affect the efficiency of the entire semiconductor photocatalytic‐based air purification.^[^ ^58^, ^59^, ^60^, ^61^ ^]^

In addition, the generation and utilization of reactive oxygen species (ROS) is a crucial process during the semiconductor photocatalysis‐based removal of air pollutants (Figure 1B).^[^ ^62^, ^63^, ^64^, ^65^, ^66^, ^67^ ^]^ In general, oxygen (O_2_, the second most abundant gas on the earth) as a moderate oxidant is considered as a “green” character with regard to active oxygen content (atom economy).^[^ ^68^, ^69^ ^]^ The excited electrons (e^–^) and holes (h^+^) will react with the O_2_ to generate different ROS (superoxide (O_2_^⋅–^), hydroxyl (⋅OH), hydrogen peroxide (H_2_O_2_), and singlet oxygen (^1^O_2_)), and the formation processes are shown in Equations (1)–(8).^[^ ^9^, ^64^, ^67^, ^68^, ^70^ ^]^ The formation potential and electron transfer of ROS production are also important factors that influenced the selection and efficiency of the photocatalysts

(1) $$O_{2}+e^{-}\to {O_{2}}^{\cdot -}$$

(2) $$h^{+}+H_{2}O/OH^{-}\to \cdot \mathrm{OH}+H^{+}$$

(3) $$2O_{2}+2H^{+}\to {O_{2}}^{\cdot -}+H_{2}O_{2}$$

(4) $${O_{2}}^{\cdot -}+2H^{+}\to H_{2}O_{2}$$

(5) $${O_{2}}^{\cdot -}+2H^{+}+e^{-}\to H_{2}O_{2}$$

(6) $$H_{2}O_{2}+e^{-}\to 2OH^{-}$$

(7) $$H_{2}O_{2}+e^{-}\to \cdot \mathrm{OH}+OH^{-}$$

(8) $${O_{2}}^{\cdot -}+2H^{+}+2e^{-}\to \cdot \mathrm{OH}+OH^{-}$$

Based on the above‐mentioned factors and requests for heterogeneous catalysis of air purification, a suitable and efficient photocatalyst shows great potential. Nevertheless, the most developed semiconductors exhibit a wide bandgap, resulting in low solar energy utilization efficiency.^[^ ^52^, ^71^, ^72^, ^73^, ^74^, ^75^ ^]^ The other photocatalysts have a high economic cost, complex synthetic process, certain toxicity, and inactivation, which remain a “bottleneck” of the photocatalysts.^[^ ^54^, ^76^, ^77^, ^78^, ^79^, ^80^ ^]^ Therefore, designing robust photocatalysts that are low‐cost, abundant, and facile in preparation, visible‐light‐responsive, efficient, sustainable, and stable in multiphase catalysis reaction is challenging. Recently, a metal‐lacking polymeric carbon nitride (PCN) has received interest in the research community, which can achieve the visible‐light‐driven water splitting with the assistance of sacrificial electrons donor/acceptor and co‐catalysts.^[^ ^45^, ^49^, ^81^, ^82^, ^83^, ^84^, ^85^ ^]^ A PCN is a linear polymer that the plane is composed of triazine and heptazine units connected by amino (–NH_2_) groups and the interlayers are stacked by van der Waals forces of covalent C–N bonds.^[^ ^45^, ^49^, ^82^, ^83^, ^84^, ^86^, ^87^ ^]^ Given the contribution of the atoms’ orbitals from the PCN, the photocatalytic redox reactions primarily occur in the carbon and nitrogen atoms.^[^ ^45^, ^49^, ^82^ ^]^ Based on previous literature, the PCN photocatalysts with heptazine ring structure demonstrate various advantages and potentials in the heterogeneous catalysis of air purification: 1) low synthesis cost and simple process can be easily achieved by thermally condensing the low‐cost, N‐prosperous and O‐lacking composites involving the core of C–N precursors (e.g., melamine (MA), dicyandiamide (DCDA), cyanamide (CA), urea (U), and thiourea (T)).^[^ ^49^, ^81^, ^82^, ^87^, ^88^ ^]^ 2) The suitable electronic band structure (2.7 eV) and band positions are satisfactory to activate the O_2_ into the O_2_^⋅–^ and overcome the Gibbs free energy, which is less than zero of the catalytic reaction.^[^ ^67^, ^89^ ^]^ 3) The redshift of light absorption of the PCN can provide more chances to absorb photons and participate in the visible‐light‐driven heterogeneous catalysis of air purification.^[^ ^90^, ^91^, ^92^, ^93^ ^]^ 4) The high physicochemical/mechanical/chemical/thermal stabilities (attributed to its aromatic C–N heterocycles) of the PCNs reduce the inactivation and photo corrosion, thereby extending the life of the photocatalysts.^[^ ^94^, ^95^, ^96^, ^97^, ^98^ ^]^ 5) A similar layered structure with graphite for PCN provides a large reaction space, improves gas absorption/desorption, and shortens the transmission distance of photogenerated charges.^[^ ^45^, ^49^, ^82^, ^99^, ^100^, ^101^ ^]^ 6) The surface amino and hydroxyl groups of the PCN can serve as defects and surface engineering sites (anchoring inorganic/organic functional monomers), and the lone pair of the N atoms can delocalize the electrons to expand the *π*‐conjugated systems.^[^ ^45^, ^102^ ^]^ 7) Theoretical calculations indicate that the Lewis basic sites of PCNs can donate electrons to the adsorbed air pollutants and decrease the bond order of N–O/C–S–C/C–H, thereby facilitating the conversion of air pollutants.^[^ ^12^, ^17^, ^103^, ^104^ ^]^ Therefore, at present, PCN is considered as a potential and transformative photocatalyst used in reforming air pollutants.

**Figure 2:**
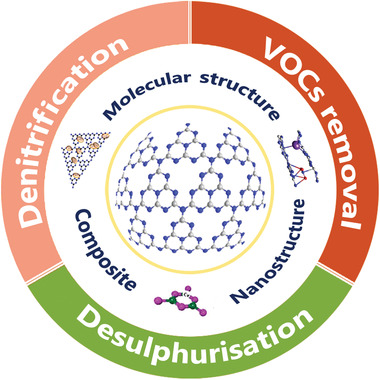
Systematic modifications of air purification using PCN‐based photocatalysts.

In addition, the pristine PCN photocatalysts have achieved success in heterogeneous catalysis of air purification, such as denitrification, desulfurization, and VOC removal.^[^ ^105^, ^106^, ^107^ ^]^ Nevertheless, due to the limited synthesis method and intrinsic polymer nature, the photocatalytic activity of pristine PCN is seriously far from the practical application because of the relatively low surface area, high exciton binding energy, sluggish charges kinetics, insufficient sunlight absorption, the low adsorption capacity of air pollutants, insufficient activation of air molecular, which greatly inhibiting its potential photocatalytic applications in air‐related revolution.^[^ ^84^, ^108^, ^109^ ^]^ Subsequently, considerable achievements have been emerged in advancing the efficiency based on PCN semiconductors in multiphase catalysis of air purification (**Figure** **2**). The specific regulation strategies of the PCNs primarily focus on structure modification based on its intrinsic nature, including nanostructure modulation, electronic structure programming, and heterostructure construction.^[^ ^95^, ^96^, ^110^, ^111^ ^]^ The nanostructure of PCN can be easily modulated because of the van der Waals force that existed in the interlayers and surface functional groups through a series of synthetic methods (e.g., exfoliation, template, and solvothermal strategies) to push the mass transfer and charge kinetics.^[^ ^112^, ^113^, ^114^ ^]^ The sufficient flexibility of the structure and polymeric nature of PCN allows control by atomic and molecular doping (introducing external impurities) to regulate the electronic structure and improve the optical absorption, and ROS generation.^[^ ^115^, ^116^, ^117^, ^118^ ^]^ The suitable bandgap and tunable band positions of PCN can be matched with other semiconductors to construct heterojunctions, and the synergistic effects can reduce charge recombination, enhance photon absorption, and accelerate mass transfer.^[^ ^50^, ^63^, ^119^ ^]^ Designing the PCN‐based photocatalysts is of great importance for diversifying and spreading the application fields of air purification. However, based on previous reports, systematic reviews about air purification by using functional PCN‐based photocatalysts are still lacking.^[^ ^49^, ^120^, ^121^, ^122^ ^]^

**Figure 3:**
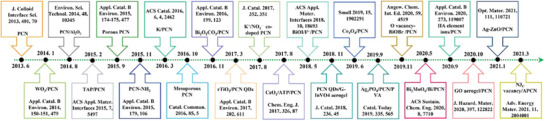
A brief timeline of the development of representative PCN‐based photocatalysts in air purification.

Herein, the brief synthetic pathways and latest research approaches for advancing PCN‐based photocatalysts and the progress of the remediating air purification‐associated issues (e.g., denitrification, desulfurization, and VOC removal) are given in the review (**Figure** **3**).^[^ ^62^, ^63^, ^105^, ^107^, ^115^, ^123^, ^124^, ^125^, ^126^, ^127^, ^128^, ^129^, ^130^, ^131^, ^132^, ^133^, ^134^, ^135^, ^136^, ^137^, ^138^, ^139^ ^]^ The structure regulation for optimizing optical absorption, charge kinetics, gas activation, adsorption of reactants/desorption of products are analyzed and discussed, which can be constructive and instructive for readers to deeply understand on structure‐activity relationship of the PCN‐based and other photocatalysts and be in turn helpful for exploring various photocatalysts. The current status to be changed, limits to be resolved, and long‐term directions to be developed for PCN‐based catalysts and composites are proposed to push the practical application in atmospheric environment remediation.

## 2 Formation of PCN Materials

### 2.1 Pristine PCN

The pristine PCN photocatalyst is commonly synthesized by thermally condensing that leads to a combination of polyaddition and polycondensation reactions of the low‐cost, N‐prosperous, and O‐lacking composites involving the core of C–N precursors (e.g., MA, DCDA, CA, U, and T).^[^ ^49^, ^81^, ^82^ ^]^

### 2.2 Functional PCN

The size, dimension, morphology, crystallinity, optical/electronic characters, chemical composition, and catalysis properties of functional PCN photocatalysts can be modulated by tailoring the nanostructure, regulating the molecular structure, and constructing the composites.^[^ ^49^, ^81^, ^82^, ^83^, ^84^ ^]^ Herein, we review the effects of exfoliation strategy, template and template‐free method, atomic and molecular doping, and composites construction on the performance of PCN‐based photocatalysts.

#### 2.2.1 Nanostructure

##### Exfoliation

Due to the stacking of the polymeric layers, the bulk PCN counterparts usually exhibit very low specific surface areas, reduced the carriers transfer, insufficient active sites, and decreased reactants adsorption, thus limiting the photocatalytic efficiency and application of PCN.^[^ ^81^ ^]^ To advance the utilization of PCN, low‐dimensional PCN with atomic or molecular size/dimension can be obtained through liquid exfoliation.^[^ ^99^, ^141^, ^142^ ^]^ It has been evidenced that the PCN nanosheets could be obtained with the assistance of organic solvents, acid, and base solutions. For instance, Yang et al. used isopropyl alcohol as a solvent and sonication‐assisted method to exfoliate the bulk PCN into ultrathin PCN nanosheets .(≈2 nm)^[^ ^142^ ^]^ In addition, the thermal‐oxidation etching route and thermal exfoliation with alkali metal salts are also extensively used to produce the low‐dimensional PCN nanomaterials. In 2012, Niu et al. directly calcinated the bulk PCN in the air to obtain the 2 nm PCN nanosheets.^[^ ^140^ ^]^ In a word, the exfoliation strategy successfully produces PCN nanosheets through breaking hydrogen bonds and van der Waals stacking in the bulk PCN and suppressing the nanosheets restacking by formatting the polar C–H bonds.

##### Template Method

With the aid of different templates (soft and hard templates), the PCN forms flexible structures and different morphologies, which are endowed with improved surface areas, active sites, and charge dynamics.^[^ ^95^, ^143^, ^144^, ^145^ ^]^ The PCN photocatalysts with mesoporous, porous, spherical, and tubular structures can be prepared by thermal condensing the mixture of silica templates (hard templates) and precursors contained carbon and nitrogen, etching by NH_4_HF_2_ (removing the silica templates). For instance, Zhang et al. used the SBA‐15 mesozeolite as the template and CA as the precursor to produce ordered mesoporous PCN.^[^ ^95^ ^]^ Sun et al. first employed core‐shell silica as the hard template to penetrate with CA, then thermal condensation to produce hollow PCN nanospheres.^[^ ^143^ ^]^ In addition, the PCN with desired porous structures and surface morphologies can be also produced through the greener soft templates (surfactants, amphiphilic block polymers, and ionic liquids).^[^ ^82^, ^146^, ^147^ ^]^ In 2010, Wang et al. used kinds of soft templates to successfully synthesize mesoporous PCN, which is endowed with high surface area and conductivity.^[^ ^146^ ^]^ Yan et al. used MA (as an unresponsive precursor) and Pluronic P123 (as a soft template) to achieve mesoporous PCN in 2012.^[^ ^147^ ^]^

##### Template‐Free Method

The supramolecular method, as a common template‐free method, is extensively used to synthesize nanostructured PCN materials (flower‐like and spherical structures) without a post‐treatment procedure for removing the template.^[^ ^82^, ^148^, ^149^, ^150^, ^151^ ^]^ The PCN with desired structure and morphologies can be controlled by tuning the monomers ratio, solvent types, temperature, precursor styles (hydrogen‐bonding donor–acceptor pairs). The key to this supramolecular method is to use the noncovalent interactions (hydrogen bonding) to form the desired ordered structural unit. For instance, the melamine mixed with cyanuric acid or trithiocyanuric acid can be used to produce ordered hollow PCN, layered PCN, PCN nanotubes through the supramolecular preorganization of hydrogen‐bonded molecular assemblies by Jun et al. and Shalom et al.^[^ ^150^, ^151^ ^]^

#### 2.2.2 Molecular Structure

The molecular structure of PCN photocatalysts can be regulated by introducing external impurities to manipulate the physical and chemical characters.^[^ ^82^, ^84^, ^152^, ^153^, ^154^, ^155^, ^156^, ^157^ ^]^ The introduction of external impurities is mainly divided into two types, including atomic doping (anions, metal cations, and co‐doping) and molecular doping (copolymerization). For instance, Liu et al. directly thermal condensing the PCN under an H_2_S atmosphere to obtain sulfur doped PCN.^[^ ^152^ ^]^ Zhang et al. thermal polymerization the mixture of the DCDA and barbituric acid to produce the organic groups anchored PCN.^[^ ^156^ ^]^ However, the atomic doping strategy may also induce some disadvantages (forming recombination center for charges) because of the broken symmetry and purities in the PCN.^[^ ^82^, ^158^ ^]^

#### 2.2.3 Composites

The PCN with flexible structure and polymeric nature endows tunable and close interconnection with other semiconductors to construct various PCN‐based composites.^[^ ^45^, ^81^, ^82^, ^86^ ^]^ The PCN‐based composites can be divided into the following types: Schottky heterojunctions, type I heterojunctions, type II heterojunctions, direct/indirect Z‐scheme heterojunctions, and S‐scheme heterojunctions. For instance, Pan et al. successfully fabricated an indirect Z‐scheme Fe_2_O_3_/reduced graphene oxide (RGO)/PCN, achieving efficient overall water splitting.^[^ ^159^ ^]^ Xia et al. designed an S‐scheme heterojunction composed of CeO_2_ and PCN, which showed highly efficient inactivation of bacteria with visible light.^[^ ^160^ ^]^

## 3 Application of PCN in Environmental Catalysis

### 3.1 PCN for Denitrification

Various types of NO, NO_2_, N_2_O, N_2_O_3_ constitute NO*_x_*, and the most (95%) of the NO*_x_* is NO.^[^ ^158^, ^159^ ^]^ The toxic NO_2_ can be formed through oxidizing NO in the atmosphere as shown in the following equations^[^ ^163^, ^164^ ^]^

(9) $$\mathrm{NO}+1/2O_{2}\to NO_{2}$$

(10) $$NO_{2}+h\nu \to \mathrm{NO}+O$$

(11) $$\mathrm{NO}+\mathrm{HC}+O_{2}+\mathrm{sunlight}\to NO_{2}+O_{3}$$

NO*_x_* is generally released from natural processes (e.g., volcanic activity and organic matter decomposition) and human activities (e.g., exhausts release and fossil fuel combustion).^[^ ^8^, ^9^, ^50^ ^]^ Respiratory diseases and even death can be triggered and resulted from breathing the air containing toxic NO*_x_*.^[^ ^7^, ^25^, ^27^ ^]^ Recently, physical adsorption (PA), catalytic redox (CR), and photocatalysis are proposed and developed for NO*_x_* elimination (denitrification).^[^ ^21^, ^25^, ^31^, ^165^ ^]^ Owing to the shortcomings of expensive cost and secondary environmental pollution in PA and CR, photocatalytic oxidation denitrification (PODN) as the most convenient method at ppb levels (indoor air concentration) arouses great interest, which is scientific, economical, and environmentally friendly.^[^ ^29^, ^103^, ^166^, ^167^ ^]^

**Figure 4:**
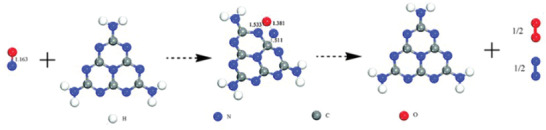
Theoretical calculations of NO decomposition on PCN. Reproduced with permission.^[^ ^103^ ^]^ Copyright 2010, Royal Society of Chemistry.

To date, various inorganic materials are developed in multiphase catalytic elimination of air pollution driven by solar energy and achievements have been made.^[^ ^29^, ^168^, ^169^, ^170^ ^]^ However, the noneconomical and nonpractical properties of the inorganic materials are far from the expectations and practical applications. PCN as a visible‐light‐responsive material was firstly applied for water decomposition by Wang et al., it became a popular and state‐of‐the‐art material for photocatalysis.^[^ ^45^ ^]^ Interestingly, Zhu et al. firstly introduced PCN into the NO decomposition under high temperature (above 400 °C), exhibiting 15.06% of NO conversion.^[^ ^103^ ^]^ Theoretical calculations indicated that the adsorbed NO could accept electrons from the Lewis basic sites of PCN and the energy of the N–O bond could be decreased, thereby promoting denitrification efficiency (**Figure** **4**). This novel discovery indicated that the PCN as a growing and potential star could be used in NO conversion, and future works should focus on stabilizing and activating the PCN for usage at the economical attraction and yield considerable denitrification performance even under mild conditions. In 2013, Dong et al. used the PCN into the visible‐light‐driven NO (ppb levels) conversion (32.1% for the optimal PCN‐240 sample after 45 min of irradiation) under ambient temperature without any sacrificial agents by adjusting the pyrolysis time of PCN materials.^[^ ^105^ ^]^ The enhancement of crystallinity and surface areas of PCN could be achieved simultaneously, which were favorable factors for promoting the PODN activity. Given the suitable band positions of the PCN, the photoexcited electrons exhibited a remarkable capacity to reduce O_2_ into O_2_^⋅–^.^[^ ^45^, ^67^, ^171^, ^172^ ^]^ The intermediate products (NO_2_) are mineralized and convert to NO_3_^–^ with the ROS or h^+^, and the possible oxidation processes of NO on PCN are shown in the following equations^[^ ^62^, ^65^, ^123^, ^173^, ^174^, ^175^ ^]^

(12) $$\mathrm{PCN}+h\nu \to e^{-}+h^{+}$$

(13) $$NO_{x}+h^{+}\to N{O_{3}}^{-}$$

(14) $$e^{-}+O_{2}\to {O_{2}}^{\cdot -}$$

(15) $${O_{2}}^{\cdot -}+2H^{+}+e^{-}\to H_{2}O_{2}$$

(16) $$H_{2}O_{2}+e^{-}\to \cdot \mathrm{OH}+OH^{-}$$

(17) $${O_{2}}^{\cdot -}+2H^{+}+2e^{-}\to \cdot \mathrm{OH}+OH^{-}$$

(18) $$\mathrm{NO}+{O_{2}}^{\cdot -}\to N{O_{3}}^{-}$$

(19) $$\mathrm{NO}+\cdot \mathrm{OH}\to N{O_{3}}^{-}$$

(20) $$2\cdot \mathrm{OH}+\mathrm{NO}\to NO_{2}+H_{2}O$$

(21) $$NO_{2}+\cdot \mathrm{OH}\to N{O_{3}}^{-}+H^{+}$$

(22) $$\mathrm{NO}+NO_{2}+H_{2}O\to 2\mathrm{HN}O_{2}$$

(23) $$2\mathrm{NO}+O_{2}+4OH^{-}+h^{+}\to 2N{O_{3}}^{-}+2H_{2}O$$

(24) $$\mathrm{NO}+h^{+}+2H_{2}O\to N{O_{3}}^{-}+4H^{+}$$

The specific formation of the intermediates during the PODN reaction on PCN is also important for exploring the reaction mechanism and process deeply, and the formation processes are shown in the following equations^[^ ^114^, ^176^, ^177^ ^]^

(25) $$\mathrm{NO}+O_{2}\to NO_{2}+N_{2}O_{4}$$

(26) $$NO_{2}\to NO_{3}+NO_{2}\to N_{2}O_{5}$$

(27) $$\mathrm{NO}+NO_{2}\to N_{2}O_{3}$$

(28) $$\mathrm{NO}+N_{\left(\mathrm{CN}\right)}\to N_{2}O$$

(29) $$\mathrm{NO}+e^{-}\to NO^{-}$$

(30) $$NO_{2}+e^{-}\to N{O_{2}}^{-}+N_{\left(\mathrm{CN}\right)}\to N_{2}{O_{2}}^{-}$$

(31) $$N_{2}{O_{2}}^{2-}+{O_{2}}^{\cdot -}\to N{O_{3}}^{-}$$

(32) $$NO^{-}+{O_{2}}^{\cdot -}\to N{O_{3}}^{-}$$

(33) $$N_{2}{O_{2}}^{2-}+\cdot \mathrm{OH}\to N{O_{3}}^{-}$$

(34) $$NO^{-}+\cdot \mathrm{OH}\to N{O_{3}}^{-}$$

Although the pristine PCN exhibits remarkable potential for its utilization in NO*_x_* abatement driven by solar energy, the practical application of PCN in PODN fields is strongly limited because of its thick layered structures, weak intermolecular van der Waals forces, or electrostatic forces existed between each layer and carbonization of either molecular or chemical precursors during the high‐temperature polymerization.^[^ ^45^, ^105^, ^178^ ^]^ In addressing the aforementioned problems, considerable effort has been made on PCN‐based photocatalysts by a nanostructure design (adjusting the polymerization process, incorporating the defective sites, controlling the morphologies, and transforming the types of the semiconductor).^[^ ^120^, ^179^, ^180^, ^181^, ^182^ ^]^ The electronic structure regulations are used to modify the light absorption of and the redox potentials through adding cations and anions and co‐doping, or molecular doping of PCN.^[^ ^115^, ^117^, ^125^, ^128^, ^136^, ^183^ ^]^ The heterostructure constructions through band alignment are also designed to accelerate the kinetics of charges, and the synergistic effects of multi‐components increase the adsorption of reactants/desorption of products, mass transfer, and ROS generation, thereby promoting the PODN efficiency.^[^ ^70^, ^184^, ^185^ ^]^ Recent progresses of PCN‐based photocatalysts with various structure modifications, which are used in POND, are shown in **Table** **1**.

**Table 1:** Summary of recently reported PCN‐based photocatalysts utilized in denitrification reaction

Photocatalysts	Types	Experimental conditions	Light source	Analyzer	Main products and NO removal ratio	Refs.
PCN (precursor regulation)	Nanostructure design	Reactor: 4.5 L Cat^a)^.: 0.1 g Conc.^b)^: 600 ppb Time^c)^: 30 min	100 W THL^d)^ (*λ* > 420 nm)	Model 42i‐TL^e)^	Products: NO_3_^–^ *η* ^f)^ (NO): 26.2% (PCNM^g)^), 32.2% (PCNU^h)^), 29.2% (PCNT^i)^), 22.2% (PCND^j)^)	^[^ ^186^ ^]^
Defective PCNT/PCNU	Nanostructure design	Reactor: 4.5 L Cat.: 0.1 g Conc.: 600 ppb Time: 30 min	150 W THL (*λ* > 420 nm)	Model 42i‐TL	Products: NO_3_^–^ *η*(NO): 39.4% (PCNT); 40.4% (PCNU)	^[^ ^187^ ^]^
PCN (precursor mass regulation)	Nanostructure design	Reactor: 4.5 L Cat.: 0.2 g Conc.: 600 ppb Time: 30 min	150 W THL (*λ* > 420 nm)	Model 42i‐TL	Products: NO_3_^–^ *η*(NO): 48.3%	^[^ ^188^ ^]^
PCN (precursor temperature and time regulation)	Nanostructure design	Reactor: 4.5 L Cat.: 0.2 g Conc.: 600 ppb Time: 30 min	150 W THL (*λ* > 420 nm)	Model 42i‐TL	Products: NO_3_^–^ *η*(NO): 32.7%	^[^ ^189^ ^]^
PCN (add water into precursors)	Nanostructure design	Reactor: 4.5 L Cat.: 0.2 g Conc.: 600 ppb Time: 30 min	150 W THL (*λ* > 420 nm)	Model 42i‐TL	Products: NO_3_^–^ *η*(NO): 48.0%	^[^ ^190^ ^]^
Porous PCN (add water into precursors, then add HCl)	Nanostructure design	Reactor: 4.5 L Cat.: 0.15 g Conc.: 600 ppb Time: 30 min	LED lamp (*λ* = 448 nm)	Chemiluminescence	Products: NO_3_^–^ *η*(NO): 71.2%	^[^ ^123^ ^]^
Porous PCN nanosheets (thermal exfoliation in the air at different temperatures)	Nanostructure design	Reactor: 4.5 L Cat.: 0.1 g Conc.: 600 ppb Time: 60 min	150 W THL (*λ* > 420 nm)	Model 42i‐TL	Products: NO_3_^–^ *η*(NO): 33.9%	^[^ ^112^ ^]^
Porous PCN (repeated thermal treatment with pristine PCN two times in the air)	Nanostructure design	Reactor: 4.5 L Cat.: 0.2 g Conc.: 600 ppb Time: 30 min	LED lamp (*λ* > 420 nm)	Model T200^k)^	Products: NO_3_^–^ *η*(NO): 65%	^[^ ^191^ ^]^
PCN (repeated thermal treatment with pristine PCN in the air)	Nanostructure design	Reactor: 4.5 L Cat.: 0.2 g Conc.: 600 ppb Time: 30 min	LED lamp (*λ* > 400 nm)	Model T200	Products: NO_3_^–^ *η*(NO): 68%	^[^ ^181^ ^]^
Porous PCN (add water with PCN during re‐thermal treatment in the air)	Nanostructure design	Reactor: 4.5 L Cat.: 0.2 g Conc.: 600 ppb Time: 30 min	150 W THL (*λ* > 420 nm)	Model 42i‐TL	Products: NO_3_^–^ *η*(NO): 51.2%	^[^ ^176^ ^]^
Porous PCN (add water and HCl into precursors)	Nanostructure design	Reactor:0.785 L Cat.: 0.05 g Conc.: 600 ppb Time: 30 min	LED lamp (*λ* = 448 nm)	Model 42i‐TL	Products: NO_3_^–^ *η*(NO): 80%	^[^ ^192^ ^]^
Porous PCN (add NH_4_HCO_3_ into precursors)	Nanostructure design	Reactor: 4.5 L Cat.: 0.05 g Conc.: 600 ppb Time: 30 min	300 W Xe lamp (*λ* > 420 nm)	Model 42i‐TL	Products: NO_3_^–^ *η*(NO): 42%	^[^ ^113^ ^]^
Porous PCN (add (NH_4_)_2_SO_4_ into precursors)	Nanostructure design	Reactor: 4.5 L Cat.: 0.2 g Conc.: 600 ppb Time: 30 min	150 W THL (*λ* > 420 nm)	Model 42i‐TL	Products: NO_3_^–^ *η*(NO): 47.1%	^[^ ^193^ ^]^
Porous PCN (add glyoxal into precursors)	Nanostructure design	Reactor: 0.325 L Cat.: 0.05 g Conc.: 15 ppm Time: 120 min	300 W Xe lamp (*λ* > 420 nm)	Testo 350	Products: NO_3_^–^ *η*(NO): 66.7%	^[^ ^194^ ^]^
Mesoporous PCN	Nanostructure design	Reactor: 4.5 L Cat.: 0.1 g Conc.: 600 ppb Time: 30 min	150 W THL (*λ* > 420 nm)	Model 42i‐TL	Products: NO_3_^–^ *η*(NO): 40.7%	^[^ ^195^ ^]^
Flower‐like PCN	Nanostructure design	Reactor: 4.5 L Cat.: 0.2 g Conc.: 600 ppb Time: 30 min	150 W LED lamp (*λ* > 400 nm)	Model T200	Products: NO_3_^–^ *η*(NO): 59.7%	^[^ ^180^ ^]^
N vacancy/porous PCN microtubes	Nanostructure design	Reactor: 4.5 L Cat.: 0.1 g Conc.: 400 ppb Time: 30 min	300 W Xe lamp (*λ* > 400 nm)	Model 42i‐TL	Products: NO_3_^–^ *η*(NO): 32.8%	^[^ ^196^ ^]^
N3_C_ vacancy/PCN	Nanostructure design	Reactor: 4.5 L Cat.: 0.4 g Conc.: 600 ppb Time: 30 min	LED lamp (*λ* > 420 nm)	Model T200	Products: NO_3_^–^ *η*(NO): 40.3%	^[^ ^177^ ^]^
N_3C_ vacancy/amorphous PCN	Nanostructure design	Reactor: 4.5 L Cat.: 0.1 g Conc.: 600 ppb Time: 30 min	150 W THL (*λ* > 400 nm)	Model T200	Products: NO_3_^–^ *η*(NO): 57.1%	^[^ ^139^ ^]^
N defect/PCN (thermal treatment PCN under H_2_)	Nanostructure design	Reactor: 0.45 L Cat.: 0.1 g Conc.: 600 ppm Time: 30 min	150 W THL (*λ* > 400 nm)	Model 42i‐TL	Products: NO_3_^–^ *η*(NO): 41.84%	^[^ ^197^ ^]^
C vacancy/PCN (mix melamine with cyanuric acid)	Nanostructure design	Reactor: 0.23 L Cat.: 0.1 g Conc.: 600 ppb Time: 30 min	300 W Xe lamp (*λ* > 420 nm)	GC‐14B^l)^	Products: N_2_ *η*(NO): 48%	^[^ ^198^ ^]^
C vacancy/PCN (calcinating PCN under CO_2_)	Nanostructure design	Reactor: 4.5 L Cat.: 0.2 g Conc.: 600 ppb Time: 30 min	150 W LED lamp (*λ* > 400 nm)	Model T200	Products: NO_3_^–^ *η*(NO): 59.0%	^[^ ^199^ ^]^
C vacancy/⋅OH/PCN	Nanostructure design	Reactor: 0.373 L Cat.: 0.2 g Conc.: 2 ppm Time: 10 min	450 W ML^m)^ (*λ* < 400 nm)	ECL‐88A^n)^	Products: NO_3_^–^ *η*(NO): 33%	^[^ ^182^ ^]^
P‐type PCN	Nanostructure design	Reactor: 4.5 L Cat.: 0.05 g Conc.: 600 ppb Time: 30 min	Xe lamp (*λ* > 420 nm)	Model 42i‐TL	Products: NO_3_^–^ *η*(NO): 80%	^[^ ^179^ ^]^
Crystalline PCN	Nanostructure design	Reactor: 0.373 L Cat.: unmarked Conc.: 2 ppm Time: unmarked	450 W ML (*λ* < 400 nm)	ECL‐88A	Products: NO_3_^–^ *η*(NO): 46%	^[^ ^200^ ^]^
PCN/Al_2_O_3_	Nanostructure design	Reactor: 4.5 L Cat.: 0.2 g Conc.: 600 ppb Time: 30 min	300 W Xe lamp (*λ* > 420 nm)	Model 42i‐TL	Products: NO_3_^–^ *η*(NO): 77.1%	^[^ ^62^ ^]^
PCN‐NH_2_ (thermal treatment under H_2_/O_2_)	Nanostructure design	Reactor: 4.5 L ( Cat.: 0.2 g Conc.: 600 ppm Time: 30 min	30 W LED lamp (*λ* > 420 nm)	Model T200	Products: NO_3_^–^ *η*(NO): 60.7% (H_2_)	^[^ ^124^ ^]^
Perfected *π*‐conjugated PCN (add NaOH, NaNO_3_, and NaHCO_3_ into precursors, respectively)	Nanostructure design	Reactor: 4.5 L Cat.: 0.1 g Conc.: 600 ppb Time: 6 min	350 W Xe lamp (*λ* > 400 nm)	chemiluminescence	Products: NO_3_^–^ *η*(NO): 53% (NaOH)	^[^ ^201^ ^]^
Crystalline CN	Nanostructure design	Reactor: 0.16 mL Cat.: 0.1 g Conc.: 2 ppm Time: 10 min	450 W ML (*λ* > 400 nm)	ECL‐88A	Products: NO_3_^–^ *η*(NO): ≈45%	^[^ ^200^ ^]^
TAP^p)^/PCN	Electronic structure regulation	Reactor: 4.5 L Cat.: 0.1 g Conc.: 600 ppb Time: 40 min	30 W LED lamp (*λ* > 420 nm)	Model T200	Products: NO_3_^–^ *η*(NO): 59.4%	^[^ ^115^ ^]^
B/PCN	Electronic structure regulation	Reactor: 4.5 L Cat.: 0.1 g Conc.: 400 ppb Time: 30 min	300 W Xe lamp (*λ* > 420 nm)	Model 42c^o)^	Products: NO_3_^–^ *η*(NO): 30.4%	^[^ ^117^ ^]^
Defective borate/PCN (NaBH_4_ treat PCN)	Nanostructure design	Reactor: 4.5 L Cat.: 0.2 g Conc.: 500 ppb Time: 30 min	300 W HL (420–700 nm)	Model 42i‐TL	Products: NO_3_^–^ *η*(NO): 56.4%	^[^ ^202^ ^]^
C/PCN	Electronic structure regulation	Reactor: 4.5 L Cat.: 0.2 g Conc.: 600 ppb Time: 30 min	100 W THL (420–700 nm)	Model 42i‐TL	Products: NO_3_^–^ *η*(NO): 50.1%	^[^ ^203^ ^]^
P/PCN	Electronic structure regulation	Reactor: 4.5 L Cat.: 0.2 g Conc.: 500 ppb Time: 30 min	150 W THL (420–700 nm)	Model 42i‐TL	Products: NO_3_^–^ *η*(NO): 42.3%	^[^ ^204^ ^]^
K/PCN	Electronic structure regulation	Reactor: 4.5 L Cat.: 0.2 g Conc.: 600 ppb Time: 30 min	150 W THL (*λ* > 420 nm)	Model 42i‐TL	Products: NO_3_^–^ *η*(NO): 44.25%	^[^ ^125^ ^]^
Ca/PCN	Electronic structure regulation	Reactor: 4.5 L Cat.: 0.2 g Conc.: 500 ppb Time: 30 min	150 W THL (*λ* > 420 nm)	Model 42i‐TL	Products: NO_3_^–^ *η*(NO): 54.78%	^[^ ^174^ ^]^
Cs/PCN	Electronic structure regulation	Reactor: 4.5 L Cat.: 0.2 g Conc.: 600 ppb Time: 30 min	150 W THL (420–700 nm)	Model 42i‐TL Column 1	Products: NO_3_^–^ *η*(NO): 51.11%	^[^ ^205^ ^]^
Sr/PCN (Sr(NO_3_)_2_ as the source of Sr)	Electronic structure regulation	Reactor: 4.5 L Cat.: 0.2 g Conc.: 600 ppb Time: 30 min	150 W THL (*λ* > 420 nm)	Model 42i‐TL	Products: NO_3_^–^ *η*(NO): 53.1%	^[^ ^206^ ^]^
Sr/PCN (multi‐site doped Sr(NO_3_)_2_)	Electronic structure regulation	Reactor: 0.785 L Cat.: 0.05 g Conc.: 600 ppb Time: 30 min	300 W Xe lamp (*λ* > 420 nm)	Model 42i‐TL	Products: NO_3_^–^ *η*(NO): 55%	^[^ ^207^ ^]^
Sr/PCN (celestite as the source of Sr)	Electronic structure regulation	Reactor: 0.785 L Cat.: 0.05 g Conc.: 600 ppb Time: 30 min	30 W LED lamp (*λ* > 420 nm)	Model 42i‐TL	Products: NO_3_^–^ *η*(NO): 67.5%	^[^ ^208^ ^]^
Group IIA element ions/PCN	Electronic structure regulation	Reactor: 0.785 L Cat.: 0.05 g Conc.: 600 ppb Time: 30 min	Xe lamp (*λ* > 420 nm)	Model 42i‐TL	Products: NO_3_^–^ *η*(NO): 62% (Ba/PCN)	^[^ ^136^ ^]^
Pd nanoparticles/PCN	Electronic structure regulation	Reactor: 4.5 L Cat.: 0.2 g Conc.: 600 ppb Time: 30 min	150 W THL (*λ* > 420 nm)	Model 42i‐TL	Products: NO_3_^–^ *η*(NO): 60.6%	^[^ ^209^ ^]^
Pd QDs^q)^/PCN	Electronic structure regulation	Reactor: 4.5 L Cat.: 0.15 g Conc.: 600 ppb Time: 40 min	LED lamp (*λ* = 448 nm)	Model T200	Products: NO_3_^–^ *η*(NO): 72%	^[^ ^210^ ^]^
O/Ba co‐doped PCN	Electronic structure regulation	Reactor: 4.5 L Cat.: 0.2 g Conc.: 500 ppb Time: 30 min	150 W THL (*λ* > 420 nm)	Model 42i‐TL	Products: NO_3_^–^ *η*(NO): ≈58%	^[^ ^211^ ^]^
O/La co‐doped PCN	Electronic structure regulation	Reactor: 4.5 L Cat.: 0.1 g Conc.: 500 ppb Time: 30 min	150 W THL (*λ* > 420 nm)	Model 42i‐TL	Products: NO_3_^–^ *η*(NO): 50.4%	^[^ ^183^ ^]^
K^+^/NO_3_^–^ co‐doped PCN	Electronic structure regulation	Reactor: 4.5 L Cat.: 0.2 g Conc.: 500 ppb Time: 30 min	150 W THL (*λ* > 420 nm)	Model 42i‐TL	Products: NO_3_^–^ *η*(NO): 40.33%	^[^ ^128^ ^]^
GO^r)^/PCN aerogel	Heterostructure construction	Reactor: 4.5 L Cat.: 0.2 g Conc.: 500 ppb Time: 30 min	150 W halide lamp (*λ* > 420 nm)	Model 42i‐TL	Products: NO_3_^–^ *η*(NO): 46.1%	^[^ ^137^ ^]^
G^s)^/MPCN^t)^ (GO/MPCN)	Heterostructure construction	Reactor: 4.5 L Cat.: 0.1 g Conc.: 600 ppb Time: 30 min	150 W THL (*λ* > 420 nm)	Model 42i‐TL	Products: NO_3_^–^ *η*(NO): 64.9% (G/MPCN), 60.7% (GO/MPCN)	^[^ ^212^ ^]^
RGO^u)^/hollow PCNS/carbonized polymer nanofibers	Heterostructure construction	Reactor: 1.6 L Cat.: 0.05 g Conc.: 600 ppb Time: 30 min	20 W lamp (*λ* > 420 nm)	Model 42i‐TL	Products: NO_3_^–^ *η*(NO): 64%	^[^ ^213^ ^]^
Ag/PCN	Heterostructure construction	Reactor: 4.5 L Cat.: 0.2 g Conc.: 600 ppb Time: 30 min	300 W THL (*λ* > 420 nm)	Model 42i‐TL	Products: NO_3_^–^ *η*(NO): 54.3%	^[^ ^214^ ^]^
Au/PCN	Heterostructure construction	Reactor: 4.5 L Cat.: 0.2 g Conc.: 500 ppb Time: 30 min	150 W THL (*λ* > 420 nm)	Model 42i‐TL	Products: NO_3_^–^ *η*(NO): 41.0%	^[^ ^65^ ^]^
Cu/PCN	Heterostructure construction	Reactor: 0.785 L Cat.: 0.2 g Conc.: 600 ppb Time: 30 min	300 W Xe lamp (*λ* > 420 nm)	Model 42i‐TL Column 1	Products: NO_3_^–^ *η*(NO): 51.0%	^[^ ^162^ ^]^
Bi/PCN	Heterostructure construction	Reactor: 4.5 L Cat.: 0.2 g Conc.: 600 ppb Time: 30 min	LED lamp (*λ* = 448 nm))	Model T200	Products: NO_3_^–^ *η*(NO): 65%	^[^ ^215^ ^]^
Bi nanoparticles/PCN	Heterostructure construction	Reactor: 4.5 L Cat.: 0.2 g Conc.: 500 ppb Time: 30 min	150 W THL (*λ* > 420 nm)	Model 42i‐TL	Products: NO_3_^–^ *η*(NO): 60.8%	^[^ ^173^ ^]^
Bi Spheres/PCN	Heterostructure construction	Reactor: 4.5 L Cat.: 0.2 g Conc.: 600 ppb Time: 30 min	150 W THL (*λ* > 420 nm)	Model 42i‐TL	Products: NO_3_^–^ *η*(NO): 59.7%	^[^ ^216^ ^]^
OPCN/K‐PCN	Heterostructure construction	Reactor: 4.5 L Cat.: 0.2 g Conc.: 550 ppb Time: 30 min	150 W THL (*λ* > 420 nm)	Model 42i‐TL	Products: NO_3_^–^ *η*(NO): 54%	^[^ ^64^ ^]^
PCNM/PCNU	Heterostructure construction	Reactor: 4.5 L Cat.: 0.1 g Conc.: 600 ppb Time: 30 min	150 W THL (*λ* > 420 nm)	Model 42i‐TL	Products: NO_3_^–^ *η*(NO): 41.3%	^[^ ^217^ ^]^
SnO_2_ QDs/PCN	Heterostructure construction	Reactor: 8.4 L Cat.: 0.4 g Conc.: 400 ppb Time: 30 min	150 W THL (*λ* > 420 nm)	Model 42i‐TL	Products: NO_3_^–^ *η*(NO): 68%	^[^ ^218^ ^]^
(BiO)_2_CO_3_/PCN	Heterostructure construction	Reactor: 4.5 L Cat.: 0.1 g Conc.: 600 ppb Time: 30 min	150 W THL (*λ* > 420 nm)	Model 42i‐TL	Products: NO_3_^–^ *η*(NO): 53.28%	^[^ ^185^ ^]^
Bi_2_O_2_CO_3_/PCN	Heterostructure construction	Reactor: 4.5 L Cat.: 0.1 g Conc.: 400 ppb Time: 30 min	300 W Xe lamp (*λ* > 420 nm)	Model 42c‐TL	Products: NO_3_^–^ *η*(NO): 34.8%	^[^ ^126^ ^]^
2D/2D BiOBr/PCN	Heterostructure construction	Reactor: 4.5 L Cat.: 0.1 g Conc.: 660 ppb Time: 30 min	100 W THL (*λ* > 420 nm)	Model 42c‐TL	Products: NO_3_^–^ *η*(NO): 32.7%	^[^ ^219^ ^]^
CeO_2_/PCN	Heterostructure construction	Reactor: 4.5 L Cat.: 0.15 g Conc.: 450 ppb Time: 30 min	300 W THL (*λ* > 420 nm)	Model 42c‐TL	Products: NO_3_^–^ *η*(NO): 35%	^[^ ^220^ ^]^
LaCO_3_OH/PCN	Heterostructure construction	Reactor: 4.5 L Cat.: 0.1 g Conc.: 400 ppb Time: 30 min	300 W Xe lamp (*λ* > 420 nm)	Model 42i‐TL	Products: NO_3_^–^ *η*(NO): 30.3%	^[^ ^114^ ^]^
Ti^3+^‐TiO_2_/PCN films	Heterostructure construction	Reactor: 0.85 L Cat.: unmarked Conc.: 400 ppb Time: 30 min	300 W Xe lamp (*λ* > 420 nm)	Chemiluminescence	Products: NO_3_^–^ *η*(NO): 25.8%	^[^ ^221^ ^]^
PCNT/PCNU	Heterostructure construction	Reactor: 4.5 L Cat.: 0.1 g Conc.: 600 ppb Time: 30 min	150 W Xe lamp (*λ* > 420 nm)	Model 42i‐TL	Products: NO_3_^–^ *η*(NO): 47.6%	^[^ ^222^ ^]^
Ni_3_(Co(CN)_6_)_2_/PCN	Heterostructure construction	Reactor: 2.26 L Cat.: 0.1 g Conc.: 600 ppb Time: 30 min	300 W Xe lamp (*λ* > 420 nm)	Model 42i‐TL	Products: NO_3_^–^ *η*(NO): 59.1%	^[^ ^223^ ^]^
Illite/PCN	Heterostructure construction	Reactor: 4.5 L Cat.: 0.05 g Conc.: 660 ppb Time: 6 min	300 W Xe lamp (*λ* > 420 nm)	Model 42i‐TL	Products: NO_3_^–^ *η*(NO): 63%	^[^ ^224^ ^]^
PdCl_2_/PCN	Heterostructure construction	Reactor: 4.5 L Cat.: 0.2 g Conc.: 1100 ppb Time: 30 min	30 W emitting diode (*λ* > 420 nm)	Model T200	Products: NO_3_^–^ *η*(NO): 65%	^[^ ^225^ ^]^
S‐TiO_2_ ^v)^/PCN	Heterostructure construction	Reactor: unmarked Cat.: 1 g Conc.: 1ppm Time: 60 min	Fluorescent lamps (*λ* > 420 nm)	CM2041, Casella	Products: NO_3_^–^ *η*(NO): 25%	^[^ ^226^ ^]^
rTiO_2_ ^w)^/PCN QDs	Heterostructure construction	Reactor: 4.5 L Cat.: 0.2 g Conc.: 600 ppb Time: 30 min	LED lamp (*λ* > 420 nm)	Model T200	Products: NO_3_^–^ *η*(NO): 37.4%	^[^ ^127^ ^]^
W_18_O_49_/PCN	Heterostructure construction	Reactor: 0.785 L Cat.: 0.05 g Conc.: 600 ppb Time: 30 min	300 W Xe lamp (*λ* > 420 nm)	Model 42i‐TL	Products: NO_3_^–^ *η*(NO): 83.55%	^[^ ^227^ ^]^
PI^x)^/PCN	Heterostructure construction	Reactor: 0.785 L Cat.: 0.05 g Conc.: 600 ppb Time: 10 min	300 W Xe lamp (*λ* > 420 nm)	Model 42i‐TL	Products: NO_3_^–^ *η*(NO): 47%	^[^ ^184^ ^]^
AgVO_3_/PCN/G aerogel	Heterostructure construction	Reactor: 2.26 L Cat.: 0.1 g Conc.: 600 ppm Time: 30 min	300 W Xe lamp (*λ* > 420 nm)	Model 42i‐TL	Products: NO_3_^–^ *η*(NO): 65%	^[^ ^175^ ^]^
2D/2D BiOIO_3_/I^–^/PCN	Heterostructure construction	Reactor: 2.26 L Cat.: 0.1 g Conc.: 600 ppb Time: 30 min	300 W Xe lamp (*λ* > 420 nm)	Model 42i‐TL	Products: NO_3_^–^ *η*(NO): 57%	^[^ ^228^ ^]^
RGO/PI/PCN	Heterostructure construction	Reactor: 0.785 L Cat.: 0.02 g Conc.: 600 ppb Time: 30 min	300 W Xe lamp (*λ* > 420 nm)	Model 42i‐TL	Products: NO_3_^–^ *η*(NO): 60%	^[^ ^70^ ^]^
Ag_3_PO_4_/Ag/PCN	Heterostructure construction	Reactor: 4.5 L Cat.: 0.3 g Conc.: 400 ppm Time: 90 min	300 W Xe lamp (*λ* > 420 nm)	SERVOPRO 4900	Products: NO_3_^–^ *η*(NO): 74%	^[^ ^229^ ^]^
Sb_2_WO_6_/PCN	Heterostructure construction	Reactor: 8.4 L Cat.: 0.05 g Conc.: 400 ppb Time: 30 min	Xe lamp (*λ* > 420 nm)	FT‐IR spectrometer	Products: NO_3_^–^ *η*(NO): 68%	^[^ ^230^ ^]^
BP/PCN‐MOF	Composite construction	Reactor: 2.26 L Cat.: 150 mg Conc.: 600 ppb Time: 30 min	Xe lamp (*λ* > 420 nm)	Model 42i‐TL	Products: NO_3_^–^ *η*(NO): 74%	^[^ ^231^ ^]^
O vacancy‐BiOBr /PCN	Composite construction	Reactor: 2.26 L Cat.: 0.1 g Conc.: 600 ppb Time: 30 min	300 W Xe lamp (*λ* > 420 nm)	Model 42i‐TL	Products: NO_3_^–^ *η*(NO): 63%	^[^ ^134^ ^]^
GO‐InVO4/PCN QDs aerogel	Composite construction	Reactor: 2.26 L Cat.: 50 mg Conc.: 600 ppb Time: 30 min	300 W Xe lamp (*λ* > 420 nm)	Model 42i‐TL	Products: NO_3_^–^ *η*(NO): 65%	^[^ ^131^ ^]^
Co_3_O_4_/PCN	Composite construction	Reactor: 2.26 L Cat.: 100 mg Conc.: 600 ppb Time: 30 min	300 W Xe lamp (*λ* > 420 nm)	Model 42i‐TL	Products: NO_3_^–^ *η*(NO): 57%	^[^ ^132^ ^]^

^a)^ Initial concentration of NO (Conc.)

^b)^ The mass of the photocatalysts (Cat.)

^c)^ Irradiation time (Time)

^d)^ Tungsten halogen lamp (THL)

^e)^ Thermo Environmental Instruments Inc., model 42i‐TL (Model 42i‐TL)

^f)^ The removal efficiency *η* (%) of pollutant was calculated as: *η* (%) = (1 − *C*/*C*_0_) × 100%

^g)^ The melamine as the precursor (PCNM)

^h)^ The urea as the precursor (PCNU)

^i)^ The thiourea as the precursor (PCNT)

^j)^ The dicyanamide as the precursor (PCND)

^k)^ Model T200, Advanced Pollution Instrumentation (Model T200)

^l)^ Gas chromatograph, GC‐14B Shimadzu Corp., Japan (GC‐14B)

^m)^ Mercury lamp (ML)

^n)^ Yanaco, ECL‐88A (ECL‐88A)

^o)^ Model 42c, Thermo Environmental Instruments Inc., Franklin, MA, USA (Model 42c)

^p)^ 2,4,6‐Triaminopyrimidine (TAP)

^q)^ Quantum dots (QDs)

^r)^ Graphene oxide (GO)

^s)^ Graphene (G)

^t)^ Mesoporous PCN (MPCN)

^u)^ Reduced graphene oxide (RGO)

^v)^ Sludges TiO_2_ (S‐TiO_2_)

^w)^ Rutile TiO_2_ (rTiO_2_)

^x)^ Perylene imides (PI).

#### 3.1.1 Research Progress of PCN in Denitrification

##### Tailoring the Nanostructure

**Figure 5:**
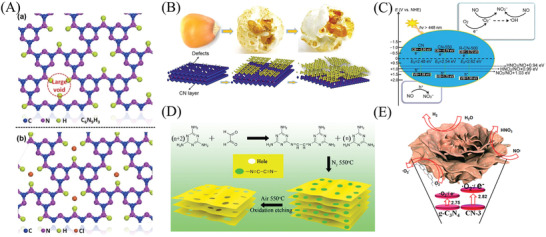
A) Schematic illustrations of a) blank C_6_N_9_H_3_ and b) the large voids padded by chloride ions of C_6_N_9_H_3_. Reproduced with permission.^[^ ^192^ ^]^ Copyright 2017, Elsevier. B) The possible formation mechanism of PCN nanosheets. C) Illustration of the electronic band structure and photocatalytic mechanism of PCNs samples. B,C) Reproduced with permission.^[^ ^191^ ^]^ Copyright 2018, Elsevier. D) Schematic formation mechanism of porous PCN. Reproduced with permission.^[^ ^194^ ^]^ Copyright 2019, Elsevier. E) Schematic mechanism of PODN on the PCN‐based photocatalysts. Reproduced with permission.^[^ ^113^ ^]^ Copyright 2016, American Chemical Society.

Tailoring the nanostructure (modulating in‐plane layers and interlayers) of PCN is performed through regulating the polymerization process (e.g., precursors mass and types, calcination time and temperature), introducing different morphologies, porous characteristics, and engineering defects into the pristine PCN based on nano templating and nano casting approach.^[^ ^49^, ^78^, ^82^, ^178^, ^179^, ^180^, ^182^, ^188^, ^189^ ^]^ The controllable nanostructure design of PCN not only improves the surface area to encourage the mass transfer kinetics but also increases the active sites on the surface of the catalysts and optimizes the energy band structure (bandgap and band position) and optical absorption, significantly advancing the efficiency of PODN. For instance, Zhang et al. synthesized different PCN polymers by calcinating various precursors, showing the diverse activity of the PODN.^[^ ^186^ ^]^ The results showed that the best precursor for the preparation of PCN was melamine, assistant with the consideration of the synthetic cost, toxicity, and products yield of PCN. This work could provide new insights and enlightenment into the PCN synthesis (selection and optimization of precursors) and an in‐depth and comprehensive cognition of the microstructure–activity relationships of PCN. A porous PCN with N defective sites could be produced by thermal treatment with urea, which showed a high specific surface area and wide bandgap, thereby promoting PODN activity.^[^ ^187^ ^]^ The enhanced activity was revealed by the increased adsorption of NO molecules favored by N defective sites, the compensated electrons favored by the production of the final products, the enhanced generation of ROS favored by the high separation of charges, and more defective sites. Moreover, the mass of precursors, along with the overlooked factor for the synthesis of PCN, exerted an unexpected effect on the nanostructure design of PCN. The nanostructure and capability of PCN semiconductors could be well‐designed and well‐tailored by varying the weight of precursors. The PCN with thin layers and increased surface area favored reaction sites and mass transfer, with elevated CB position favored improved redox ability, with promoted PODN capability were triumphantly accomplished as declining the mass of thiourea.^[^ ^188^ ^]^ The regulation of the calcination temperature and time to produce PCN exhibited similar positive effects on PODN.^[^ ^189^ ^]^ Furthermore, a porous honeycomb structure for PCN could be achieved by adding a certain amount of water into precursors before calcination, which exhibited a highly enhanced NO removal ratio (48%) compared with the pristine PCN (30.6%).^[^ ^190^ ^]^ Adding further HCl to the prepared PCN precursors, the activity of PODN for the obtained porous PCN (80%) was enhanced.^[^ ^192^ ^]^ This positive effect was due to the changed modal of thermal condensation and the introduction of Cl^–^/NH_4_^+^ and large voids (donated or accepted electrons, **Figure** **5**A), which increased the surface area and eliminated part of the quantum confinement effect, thereby enhancing the PODN activity.

In addition, porous PCN nanosheets could be obtained by thermal exfoliation, induced band structure variation and the quantum confinement effect favored by the reduced particle size associated with a decreased layer thickness.^[^ ^112^, ^181^ ^]^ For instance, Wu et al. obtained porous PCN nanosheets via a two‐step layer‐by‐layer thermal exfoliation, increased electrical conductivity and enlarged PCN bandgap (Figure 5B).^[^ ^191^ ^]^ The promoted kinetics of charges and oxidation capability could be achieved through the above structure optimization, thereby providing new chances for regulating the PCN synthesis and redox capability toward efficient PODN (Figure 5C). Organic molecules (glyoxal), as a soft template, could be introduced in the synthesis process of porous PCN nanosheets, which showed enhanced O_2_ activation and PODN activity (Figure 5D).^[^ ^194^ ^]^ The inorganic salt of (NH_4_)_2_SO_4_ and NH_4_HCO_3_ as bubble soft templates was also selected to design the porous structure of PCN.^[^ ^113^, ^193^ ^]^ These bubble soft templates endowed PCN with a porous structure and enhanced specific surface area favored gas diffusion and transfer, meanwhile, rendered up‐shifted CB position favored charge separation and transportation (Figure 5E).^[^ ^113^ ^]^ Given these benefits and advantages from the modifications of the thermal exfoliation and soft templates, the improved PCN with porous structure endows promoted performance toward PODN.

In addition, the PCN with a mesoporous structure is also widely used for PODN reaction at the ppb level.^[^ ^195^ ^]^ The mesoporous/nanosphere SiO_2_ was a type of hard template, which modifies the morphology and texture of PCN by Dong et al. The significant enhancement of activity for mesoporous PCN was ascribed to the co‐contributions of boosted surface area and pore volume, enhanced optical‐capturing ability, increased redox capability, and inhibited recombination of charge carriers. The flower‐like PCN was assembled from holy nanosheets by the co‐production of N vacancies, demonstrating a much higher visible activity of oxidation denitrification (ODN) compared with the bare PCN.^[^ ^180^ ^]^ The improved PODN efficiency of flower‐like PCN was due to the broadened absorption capability of visible light and strengthened kinetics of solar‐induced charges favored by condensing the stacking of the *π*–*π* layer and breaking the interplanar hydrogen bonds.

Powdery PCNs exhibit high activity in PODN reactions.^[^ ^9^ ^]^ However, its practical application is still limited, because the powdery catalyst can be facile blown away and lost, with time‐consuming filtration/separation.^[^ ^62^ ^]^ Therefore, the introduction of the supported technology can solve the key technical problem of the powdery photocatalyst from the test to the application stage, which provides the possibility for the powdery photocatalyst to create greater economic and environmental benefits in the actual production and application process. Firstly, the PCN was in situ immobilized on structured ceramic foam (Al_2_O_3_) by the chemical interaction, which was utilized to decomposed NO in the air under the irradiation of real indoor energy‐saving lamps.^[^ ^62^ ^]^ The photocatalytic NO removal ratio of immobilized PCN could reach up to 77.1% at a pyrolysis temperature of 600 °C, which showed superior reusability in PODN reaction. The immobilization of PCN on Al_2_O_3_ was confirmed and expected to realize practical application to remediate atmospheric environmental issues.

##### Introduction of Vacancies

The production of thin‐layered PCN in assistance with a porous structure can be achieved through the exfoliation of bulk PCN, including liquid‐phase and gas‐phase exfoliation.^[^ ^49^, ^82^, ^112^, ^181^, ^193^ ^]^ The superiority and advantages of the resultant thin‐layered PCN can be associated with the enlarged specific surface area (favored mass transportation kinetics and reactive sites) and improved electron and hole separation/transportability efficiency. In addition, the production of N defective sites in PCNs increases the adsorption of NO molecules, which leads to NO activation and conversion, thereby providing great potential for PODN.^[^ ^197^ ^]^ However, PCN materials synthesized by traditional thermal condensation usually exhibit a low density of defective and active sites, thereby leading to sluggish charge migration and PODN efficiency.^[^ ^45^, ^49^, ^82^ ^]^ Absorbing the reactants on the surface of the “perfect” semiconductor photocatalyst without any functional groups or defective sites is difficult, leading to the difficulty of photocatalytic reaction.^[^ ^232^, ^233^, ^234^ ^]^ Therefore, defect engineering on the surface of PCN materials (C/N vacancies produced on the periodic heptazine units) is considered and confirmed as an advanced modification toward efficient and stable PODN, because defects usually endow strong effects on optimizing the structure of semiconductors.^[^ ^177^, ^199^ ^]^

Moreover, the introduction of vacancies changes the surface or internal structure of the catalyst to generate new levels, regulates the photo‐electrochemical nature of the PCN catalyst, and provides enhanced reaction sites for the reactants, thereby improving the performance of the photocatalytic reaction.^[^ ^197^, ^198^ ^]^ Although vacancies as general defects exist in the semiconductors, the mediators' function of vacancies can offer new mechanisms and insights to modulate the electronic structure and charge kinetics of the photocatalysts.^[^ ^235^, ^236^, ^237^ ^]^ In general, vacancies can be generated after lattice atom escape and classified into anionic and cationic vacancies.^[^ ^238^, ^239^, ^240^, ^241^, ^242^ ^]^ The anionic vacancies are vital for multiphase catalysis because of the endowed optical‐absorbing capabilities, the function of specific adsorption/reaction sites for reactant molecules, and the capture sites for electrons.^[^ ^199^ ^]^ For example, the O vacancies as a representative anionic vacancy in metal oxides have been actively pursued in capturing electrons, activating O_2_, and utilizing solar light.^[^ ^238^, ^240^ ^]^ Based on the discovery linked to O‐vacancy inorganic materials, anionic vacancies produced from PCN could effectively adsorb/activate target air pollutants, harvest photogenerated electrons, and provide specific sites.^[^ ^177^, ^196^ ^]^ Current approaches developed for generating anionic vacancies into PCN primarily involve calcination under kinds of atmospheric conditions or at diverse temperatures.^[^ ^177^, ^196^ ^]^ The roles of anionic vacancies in spatially distributing charges, accelerating charge kinetics and activating target reactants (O_2_ and NO*_x_*) are investigated and expounded.

**Figure 6:**
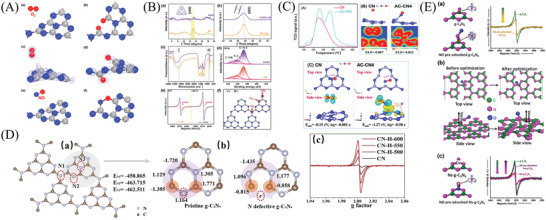
A) Models of O_2_ adsorbed on: a) PCN and b) N‐deficient PCN after geometry optimization. The corresponding calculated EDD diagrams of O_2_ absorbed on c) PCN and d) N‐deficient PCN. Reproduced with permission.^[^ ^196^ ^]^ Copyright 2019, American Chemical Society. B) a,b) XRD patterns of the obtained CN and ACN_3_ samples, and c) the comparison of the FTIR spectra, and d) N 1s XPS spectra, e) ESR spectra, and f) the comparison of schematic atomic model of incomplete ACN constructed from melon units with three kinds of nitrogen labeled as N2_C_, N3_C_, and NH*_x_*. Reproduced with permission.^[^ ^139^ ^]^ Copyright 2021, Wiley‐VCH. C) NO‐TPD spectra, electron localization function (ELF) and optimized NO adsorption of PCNs samples. Reproduced with permission.^[^ ^177^ ^]^ Copyright 2020, Elsevier. D) a) Structure of containing N‐defective PCN. b) Bader effective charge analysis. c) EPR spectra of samples. Reproduced with permission.^[^ ^197^ ^]^ Copyright 2020, Elsevier. E) The effect of the pre‐adsorption of NO on the EPR spectrum of PCN and Ns‐PCN. Reproduced with permission.^[^ ^198^ ^]^ Copyright 2017, Elsevier.

For example, Wang et al. in situ synthesized a sort of N‐vacancy PCN with porous microtubes via soft‐chemical methods.^[^ ^196^ ^]^ The surface N vacancies as specific sites functioned as capturing solar‐induced electrons and activating O_2_ while enhancing the solar‐absorbing capability of PCN (**Figure** **6**A). Moreover, the reactants diffusion and directed charge transfer were facilitated by the porous and tubular architectures of the PCN microtubes. The prepared porous PCN microtubes with N vacancies exhibited advanced NO*_x_* degradation in comparison with bulk PCN with visible light. The unique N3_C_ vacancies in the amorphous PCN matrix were stable and highly efficient during the procedure of PODN, which were ascribed to the boosted ROS generation (^1^O_2_ and O_2_^⋅−^) and improved light absorption for complete NO removal (Figure 6B).^[^ ^139^ ^]^ The PCN with three coordinated (N3_C_) N vacancies could also be achieved by directly condensing the hybrid of N‐abundant precursors (azodicarbonamide and melamine).^[^ ^177^ ^]^ Different from the pristine PCN, the obtained PCN with N vacancies showed better PODN activity and stability because of the wide absorption range of visible light and the accelerated charge transmission. The N vacancies produced in PCN could serve as the reactive sites, which weakened the adsorption of intermediates/final products, strengthened the desorption of final products, facilitated the O_2_ activation and ROS conversion (Figure 6C), thereby contributing the enhanced PODN performance and the retained reusability. When the PCN was heated under an H_2_ atmosphere, N defects were introduced, and the accelerated NO^+^ intermediate was formed to improve the PODN efficiency (Figure 6D).^[^ ^197^ ^]^ The acidic groups (e.g., SO_4_^2−^ and PO_4_^3−^) can intensify the adsorption of O_2_ and accelerate the separation of charge carrier and generation of ROS favored by hot electrons utilization, which is necessary for PODN.^[^ ^243^, ^244^ ^]^ When the NaBH_4_ is introduced into the PCN, the N defect and acidic group (borate) were simultaneously produced.^[^ ^202^ ^]^ The resultant defective borate‐decorated PCN showed high efficiency in the ppb‐level PODN reaction because of the promoted visible‐light absorbance, accelerated charge kinetics, and improved ROS generation.

Moreover, PCN with carbon vacancy was investigated and applied in PODN, showing an extraordinarily NO removal rate in comparison with pristine PCN.^[^ ^199^ ^]^ This excellent activity was ascribed to the facilitated transfer of electrons favored by the negatively shifted potential of CB, increased adsorption/activation of O_2_ molecules favored by the superior surface area and boosted electrons. In particular, the ultrathin PCN layers with abundant surface C vacancies could be obtained through thermally condensing the hybrid of melamine and cyanuric acid, demonstrating higher efficiency and stability of PODN.^[^ ^198^ ^]^ Different from the above‐mentioned conversion and decomposition mechanism, the NO was reduced into the N_2_ for the ultrathin PCN with C vacancies. The ultrathin PCN structures possessed a unique surface structure (C vacancies) that could serve as traps of the electrons to localize the photogenerated charges and function as an adsorption site for NO*_x_* through the binding of the PCN interface (Figure 6E). The direct transmission of electrons from the vacancy of PCN to the absorbed NO molecules resulted from the localization of the solar‐generated electron and NO molecule, thereby contributing to the NO reduction into N_2_. This work is important to current research work. In general, NO_2_ and NO_3_^–^ are the currently accepted products for PODN by the public, whereas the gaseous N_2_ as another clean and possible product can straightforwardly desorb from the reactive sites of PCN, thereby reducing the deactivation of photocatalysts and avoiding secondary environmental pollution.^[^ ^238^ ^]^ However, the green product of N_2_ cannot easily be observed and obtained from the NO conversion because of the high dissociation energy of the N≡O bond (632 kJ mol^–1^).^[^ ^238^ ^]^ Therefore, exploring novel photocatalysts that can be used in NO*_x_* photoreduction is of great importance.

##### Regulating Molecular Structure

**Figure 7:**
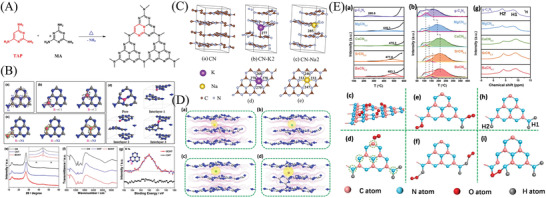
A) Incorporation of TAP into the Network of PCN. Reproduced with permission.^[^ ^115^ ^]^ Copyright 2015, American Chemical Society. B) a) Pristine PCN mode. b) Boron atom substitution doping at c) two inequivalent carbon sites or d) three inequivalent nitrogen sites. d) Interstitial doping at pore or interlayers. All samples underwent a subsequent geometry optimization. e) XRD patterns (the small inset represents the (002) peaks of all samples after enlargement) and f) FT‐IR spectra of CN, CNT, and BCNT. g) XPS high‐resolution spectra of B 1s for BCNT and CNT. Reproduced with permission.^[^ ^117^ ^]^ Copyright 2018, Elsevier. C) Calculated crystal structures of a) CN, b) CN‐K2, and c) CNNa2. d,e) Top views of the doped layer in (b) and (c), correspondingly. Reproduced with permission.^[^ ^125^ ^]^ Copyright 2016, American Chemical Society. D) DFT calculates the electron density distribution of various metals doped in PCN. E) a) NO‐TPD spectra and b) O_2_‐TPD spectra of PCN and c) M/PCN‐0.2, O_2_ physical adsorption of PCN, d) schematic diagram of possible adsorption sites for O_2_, e) O_2_ forming covalent bonds with functional groups, f) O_2_ forming hydrogen bonds with functional groups, g) ^1^H solid‐state nuclear magnetic resonance (NMR) spectra of PCN and M/PCN‐0.2, h) the H atoms in solid‐state NMR spectra, and i) O_2_ forming covalent bonds with the functional groups. Reproduced with permission.^[^ ^136^ ^]^ Copyright 2020, Elsevier.

Regulating the electronic structure (energy‐band configuration and bandgap) has been proven to be an ideal and effective strategy to determine the optical absorption ability and redox capacities, contributing to the investigation and advance of visible‐light‐sensitive PCN with optimized performance.^[^ ^49^, ^81^, ^82^, ^84^ ^]^ Adjusting and perfecting the conventional systems and electronic structure of the PCN is necessary because of the insufficient absorption and utilization of sunlight.^[^ ^45^, ^49^ ^]^ Therefore, copolymerizing another similar aromatic structure with PCN precursors as a type of molecular doping strategy is viewed as a noteworthy step to extend the electrons of the aromatic system and modify the electronic band structure of PCN.^[^ ^49^, ^82^, ^84^ ^]^ Ho et al. developed a copolymerization method, in which the monomer of the 2,4,6‐triaminopyrimidine (TAP) was copolymerized into the matrix of PCN, and the tubular PCN was produced through rolling‐up the nanosheets (**Figure** **7**A).^[^ ^115^ ^]^ The *π*‐electron conjugated units of PCN were facilitated and enlarged by the introduction of the C_4_N_2_ ring from the TAP molecules into the PCN structure. Moreover, the resultant PCN photocatalysts displayed a tunable bandgap and efficient charge kinetics, which resulted in outstanding performance toward the removal of NO gas pollutants. Zhu et al. constructed a perfected *π*‐conjugated PCN by coordination among 3p orbits of Na (NaOH) and lone electron of N 2p of PCNs with melon structure.^[^ ^198^ ^]^ The improved *π*‐conjugated PCN structure enhanced the seizing capability of visible light, boosted the activation of O_2_ favored by several active sites, and promoted the directional transfer of charges, thereby demonstrating superior activity toward PODN reaction.

Atomic doping is extensively proved and investigated to be another vital method in manipulating the electronic structure, primarily concentrating on incorporating external impurities into the photocatalysts.^[^ ^49^, ^82^, ^84^, ^245^ ^]^ Various atoms, including metal, nonmetal elements, and combinations, are incorporated into the matrix of PCN to achieve sufficient PODN performance.^[^ ^117^, ^125^, ^136^, ^183^ ^]^ For instance, phosphorus‐doped PCN (P/PCN) with a typical optical property and the down‐shifted VB‐edge of P/PCN‐5 exhibited stronger oxidation ability than that of the unmodified PCN, which ensured efficiently photocatalytic NO removal.^[^ ^204^ ^]^ The hollow B‐doped PCN tubes were produced by calcining the hybrid of the supramolecular self‐assembled precursors and boric acid, which widened the harvesting ability of visible light and advanced separation and transferability of light‐induced electron–hole pairs, thereby exhibiting the highest NO removal rate (30.4%) compared with the bulk PCN (20.8%) and PCN hollow tubes (22.9%, Figure 7B).^[^ ^117^ ^]^ Additionally, self‐doping is also developed to form some defects or vacancies by removing some elements of the photocatalyst and adjusting the energy‐band configuration and bandgap of semiconductor photocatalysts.^[^ ^158^, ^246^ ^]^ For instance, the carbon self‐doped PCN (substituted the bridging N atoms) could be produced by using a porous carbon foam template, which displayed enlarged surface area, extended near‐infrared light absorption and advanced electron–hole movement and transfer.^[^ ^203^ ^]^ The optimized carbon‐doped PCN demonstrated a remarkably high performance toward the PODN in air and exceeded other nonmetal‐doped inorganic materials including Ti‐based and Bi‐based semiconductors.

Semiconductors loaded with metals (e.g., K, Cs, Mg, Ca, Sr, Ba, and Pd) by introducing the impurities into the PCN matrix can effectively lower the barrier of the rate‐determining step and mostly inhibit the production of the toxic intermediate (NO_2_), thereby decreasing the secondary environmental pollution and improving PODN activity.^[^ ^125^, ^138^, ^205^, ^210^ ^]^ For instance, Xiong et al. expounded the group IA elements (e.g., K and Na) by doping into the PCN under the sample conditions, investigated the electronic structure and PODN activity with the assistance of experimental and theoretical combination.^[^ ^125^ ^]^ Consequently, K‐intercalated PCN exhibited higher visible PODN performance than that of Na‐doped PCN. This variance was the intercalated types of K atoms (bridging interlayer) and Na atoms (introducing into the plane) doped PCN; thus, the K/PCN could provide a delivery channel via bridging the layers, and the Na/PCN increased the recombination of charges because of the promotion of in‐planar electron density (Figure 7C). The group IIA elements (e.g., Mg, Ca, Ba, and Sr) incorporated into the PCN matrix could also provide interlayer electron channels by the formation of coordinate bonds (connecting two adjacent layers with N atoms in the triazine rings of PCN), thereby distorting the PCN structure (Figure 7D,E).^[^ ^136^, ^174^, ^206^, ^207^, ^208^ ^]^ The surface electronic delocalization was destroyed by the distorted PCN structure, thereby suppressing surface charges recombination and improving the electron transport between two layers. In addition, the functional groups were enhanced by the distorted PCN structure, providing more chemisorption sites for O_2_. Given the above‐mentioned advantages, the PCN coupled with group IIA elements exhibited superior PODN efficiency compared with the pure PCN. The transition metal (e.g., Pd nanoparticles and Pd quantum dots (QDs)) doped in the PCN also showed a similar positive effect on visible PODN reaction.^[^ ^200^, ^210^ ^]^

Co‐doping can combine the advantages of single dopants, such as the delocalized electrons and the random transmission of charge in planes, thereby retarding a high charge‐recombination rate and probability.^[^ ^128^, ^183^, ^211^ ^]^ The interlayer energy barrier of PCN could be lowered by intercalating K^+^ and NO_3_^–^ into the interlayer to build a bidirectional electronic transmission channel.^[^ ^128^ ^]^ Abundant electrons could be offered to accelerate the O_2_ activation and ROS production to endow the K^+^ and NO_3_^–^ co‐doped PCN with great visible‐light performance of ODN. This positive result was also evidenced by Cui et al. and Chen et al., and double elements co‐functionalized PCN as an electronic trapping adjuster and mediator to encourage the astringent and localization of intralayer‐delocalized electrons, thereby facilitating the adsorption and activation of gas molecules, expediting the spatial charge kinetics, suppressing toxic NO_2_ generation.^[^ ^183^, ^211^ ^]^

##### Constructing Composites

**Figure 8:**
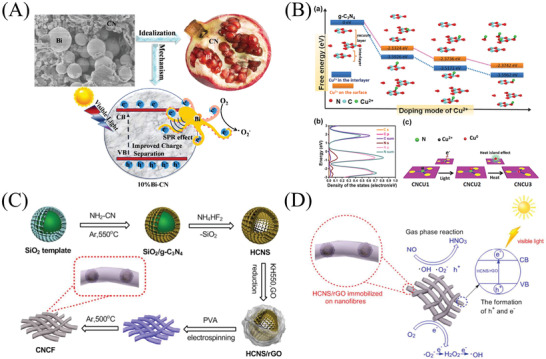
A) Proposed mechanism of the photocatalytic oxidation of NO for the 10% Bi/PCN composite. Reproduced with permission.^[^ ^215^ ^]^ Copyright 2017, Elsevier. B) Free energy of several possible modes of a) Cu^2+^ doping PCN, b) DOS of the PCN, and c) the schematic formation process of various Cu/PCN‐x samples. Reproduced with permission.^[^ ^162^ ^]^ Copyright 2019, American Chemical Society. C) The schematic production process to fabricate RGO/HPCNS. D) Schematic of the reaction mechanism for NO removal. C,D) Reproduced with permission.^[^ ^213^ ^]^ Copyright 2016, Royal Society of Chemistry.

Engineering Schottky heterojunctions by incorporating plasmonic metal nanoparticles (e.g., Au, Ag, Cu, and Bi) in photocatalysts have been developed and proven to be a potential way to efficiently decrease the recombination of the light‐excited charges and advance the charge transmission, because of their viability and efficiency for the spatial separation of charges.^[^ ^247^, ^248^, ^249^ ^]^ For instance, Li et al. used NaBH_4_ as a reducing agent to fabricate monodisperse Au nanoparticle‐supported PCN, exhibiting an enhanced NO oxidation and NO_2_ inhibition ability.^[^ ^65^ ^]^ The promotion could be attributed to the enhanced adsorption/activation of gas molecules favored by the surface plasmon resonance (SPR) Au metal site, the improved separation efficiency of photogenerated charges, the lowered rate‐determining barrier favored by the unique electronic structure of Au/PCN, and the suppressed production of the toxic intermediate (NO_2_). The semimetal bismuth (Bi) can work as a potential substitute for noble SPR metals because of its low price, easy availability, and conductive properties.^[^ ^250^, ^251^, ^252^ ^]^ Bi can also work as an excellent and stable cocatalyst to lower the overpotential and advance the separation of the charge carriers.^[^ ^253^, ^254^ ^]^ When Bi, like nanospheres or nanoparticles, was introduced into the PCN nanosheets, the novel nanocomposites exhibited highly enhanced ODN activity and stability with visible light (**Figure** **8**A).^[^ ^173^, ^215^, ^216^ ^]^ This enhanced photocatalytic performance of Bi/PCN could be attributed to the co‐contributions of the enhanced optical absorption and the advanced kinetics of charge carriers because of the SPR effects of Bi‐metal and the Schottky heterojunctions production. The enhanced light‐driven e^–^ could also reduce O_2_ to H_2_O_2_ and further convert into ⋅OH by trapping an electron, thereby further improving the NO*_x_* photooxidation efficiency. Different from a one‐step‐modified PCN photocatalyst, Zhao et al. explored the PCN photocatalyst modified by copper through multistep modifications (Figure 8B).^[^ ^162^ ^]^ The obtained Cu/PCN photocatalysts could also improve NO removal activity by increasing electron transfer and ROS generation.

The PCN‐based heterojunctions constructed with other semiconductors also exhibit accelerated kinetics of photogenerated charges, which improves carrier transfer across the junction favored by the differences in their band structures and provides the space charge separation/transport region favored by the band bending.^[^ ^49^, ^81^, ^82^, ^110^, ^255^, ^256^ ^]^ PCN coupled with graphene‐based materials (*π*‐conjugated structure and superior electric property) has been focused on improving its conductivity and catalytic performance by enhancing carrier separation/transfer and electron mobility.^[^ ^56^, ^257^, ^258^, ^259^ ^]^ The graphene/mesoporous PCN (G/MPCN) and graphene oxide/mesoporous PCN (GO/MPCN) nanocomposites endowed extremely boosted visible‐light‐driven ODN efficiency, which was caused by the enlarged surface nanostructure (accelerating the mass transfer kinetics), the improved the utilization of visible light, the boosted the reduction potential of electrons, and the encouraged the kinetics of charge carriers.^[^ ^212^ ^]^ The hollow porous PCN nanosphere (HPCNS) coupled with reduced GO (RGO/HPCNS) also exhibited a NO removal ratio of 64% (600 ppb level) and high stability because of the hollow porous morphology of HPCNS and the grafted RGO on the surface (Figure 8C,D).^[^ ^213^ ^]^

**Figure 9:**
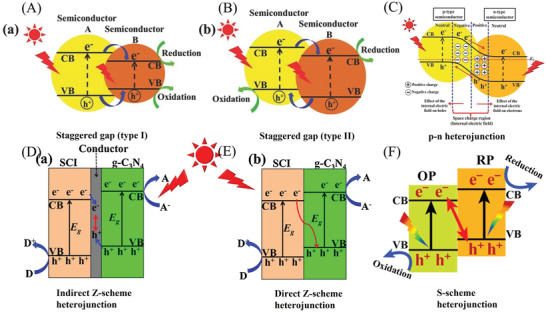
Schematic illustration of the separation of electron–hole pairs for A) type I heterojunction, B) type II heterojunction, C) p–n heterojunction. A‐C) Reproduced with permission.^[^ ^260^ ^]^ Copyright 2017, Wiley‐VCH. D) indirect Z‐scheme heterojunction, E) direct Z‐scheme heterojunction. D,E) Reproduced with permission.^[^ ^262^ ^]^ Copyright 2018, Elsevier. F) S‐scheme heterojunction. Reproduced with permission.^[^ ^263^ ^]^ Copyright 2020, Elsevier.

**Figure 10:**
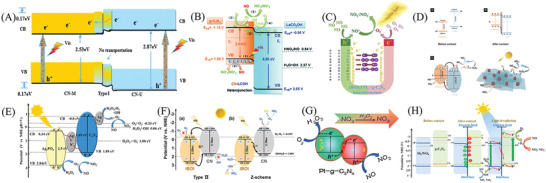
A) Illustration charge transfer of type I PCNM/PCNU heterostructures. Reproduced with permission.^[^ ^217^ ^]^ Copyright 2015, Royal Society of Chemistry. B) The proposed photocatalytic mechanism of LaCO_3_OH/PCN heterojunctions for NO degradation with visible light. Reproduced with permission.^[^ ^114^ ^]^ Copyright 2017, Royal Society of Chemistry. C) Photocatalytic mechanism of NO removal under visible‐light irradiation by NICO/PCN‐100: a) before contact, b) after contact, and c) the p–n heterojunction. Reproduced with permission.^[^ ^185^ ^]^ Copyright 2020, Elsevier. D) The proposed photocatalytic mechanism of (BiO)_2_CO_3_/PCN heterojunctions for NO degradation with visible light. Reproduced with permission.^[^ ^223^ ^]^ Copyright 2021, American Chemical Society. E) The mechanism of photocatalytic NO removal of Ag_3_PO_4_/Ag/PCN under visible light. Reproduced with permission.^[^ ^229^ ^]^ Copyright 2021, Elsevier. F) a) Type II heterojunction mechanism and b) Z‐scheme photocatalytic mechanism of NO removal under visible‐light irradiation by 30% BiOIO_3_/I^–^/PCN. Reproduced with permission.^[^ ^228^ ^]^ Copyright 2020, Elsevier. G) Photocatalytic mechanism of PI/PCN. Reproduced with permission.^[^ ^184^ ^]^ Copyright 2016, American Chemical Society. H) S‐scheme photocatalytic mechanism for Sb_2_WO_6_/PCN nanocomposite. Reproduced with permission.^[^ ^230^ ^]^ Copyright 2021, Elsevier.

Except for the PCN heterojunction constructed from graphene‐based materials, heterojunctions constructed by other semiconductors can also advance the kinetics of charges in the spatial and built‐in electric field.^[^ ^49^, ^81^, ^82^, ^110^, ^255^, ^256^, ^260^ ^]^ Band alignment is one of the keys to construct a suitable and efficient heterojunction.^[^ ^109^, ^261^ ^]^ In general, the common heterojunction types can be primarily divided into the following types: type I heterojunction, type II heterojunction, p–n heterojunction, Z‐scheme heterojunction, and S‐scheme heterojunction based on the charge transfer among different energy bands (**Figure** **9**).^[^ ^110^, ^260^, ^262^, ^263^ ^]^ For the type I heterojunction, the electrons and holes generated by light excitation migrate from a high‐potential semiconductor B to the same semiconductor A with relatively low potential. (Figure 9A).^[^ ^110^, ^234^, ^260^ ^]^ This type of migration causes a relatively suppressed separation of light‐excited carriers, but the charge kinetics of the type I heterojunction can be effectively regulated by adjusting the movement rates of charges. For instance, Dong et al. successfully and thermally treated the blended precursors of melamine and urea by in situ creating type I PCNM/PCNU metal‐free isotype heterostructures in view of staggered and aligned bands.^[^ ^217^ ^]^ The intrinsic shortcoming of charge kinetics of pristine PCN was decreased and overcome by the creation of type I PCNM/PCNU heterostructures, demonstrating excellent PODN activity and reusability at ppb‐level NO in comparison with PCN counterpart (**Figure** **10**A).

For type II heterojunctions, the high‐potential semiconductors A of photogenerated electrons that are excited by light migrate to the low‐potential semiconductors B, and the photogenerated holes are opposite, which is ascribed to the staggering energy levels between semiconductors (Figure 9B).^[^ ^110^, ^260^, ^262^, ^263^ ^]^ This type of migration as one of the potential strategies can improve photocatalytic activity by constructing a spatial and built‐in field, thereby effectively separating photogenerated carriers between semiconductors. For instance, Tian et al. in situ fabricated CeO_2_/PCN hybrid materials by a facile co‐pyrolysis of the precursors of Ce‐based and N‐abundant materials.^[^ ^220^ ^]^ The CeO_2_/PCN composite catalysts possessed brilliant PODN efficiency, which was resulted from the construction of CeO_2_/PCN type II heterojunction (endowing advanced charge kinetics across the intimate phase interfaces). Wang et al. controllably fabricated a novel heterostructure of hierarchical rouleaux triangle LaCO_3_OH/PCN nanocomposite by a hydrothermal strategy.^[^ ^114^ ^]^ During the hydrothermal process, the PCN served as a structural direct mediator and CO_3_^2–^ acted as source significantly affected the morphology manufacturing of LaCO_3_OH. The booming interfacial charge kinetics was one of the dominant factors for the improvement of LaCO_3_OH/PCN toward PODN reaction (Figure 10B). In current years, visible‐light‐responded Bi‐based catalysts with lone‐pair distortion of Bi 6s orbitals and hybridization of O 2p and Bi 6s orbitals shows the unique electronic structure, good chemical stability and controlled morphology, which have drawn increasing attention.^[^ ^264^, ^265^, ^266^ ^]^ For instance, the Bi_2_O_2_CO_3_/PCN and layered BiOBr/PCN heterojunctions, and (BiO)_2_CO_3_ nanosphere‐decorated PCN composite heterostructure demonstrated the promoted adsorption/activation of NO molecules and accelerated spatial charge separation (Figure 10C); the O_2_ molecule tended to adsorb on the electron‐rich areas and convert into O_2_^⋅–^ radicals, thereby contributing to the efficient PODN of NO.^[^ ^123^, ^185^, ^219^ ^]^

The construction of PCN‐based p–n heterojunctions is also widely considered and proved to be one of the strategies to improve the dynamics of photogenerated carriers.^[^ ^110^, ^234^, ^260^ ^]^ As shown in Figure 9C, electrons (p‐type semiconductors) excited by light tend to migrate to the CB of n‐type semiconductors, while light‐excited holes are the opposite. This type of carrier migration generates an internal electric field (IEF) in the internal space, which accelerates the separation and migration of charges, resulting in higher dynamic efficiency than the type II heterojunction. For instance, Wang et al. successfully created a composite consisted of hollow Ni–Co bimetal oxide and layered PCN through the method of low‐temperature annealing.^[^ ^223^ ^]^ A spatial conductive trend caused by the creation of p–n heterojunction based on the Ni–Co bimetallic oxide and PCN advanced the charge dynamics; and the gas circulation was promoted by the pores of the hollow Ni–Co bimetallic oxide, thereby degrading the NO with visible light and showing predominant reusability (Figure 10D).

In general, the above‐mentioned heterojunction can effectively separate light‐excited electrons and holes, thereby promoting PODN activity. However, the redox process that occurs in the above‐mentioned heterojunction is at a relatively low potential because of the specific charge transfer pathways, which sacrificed the partial redox ability of the semiconductors.^[^ ^110^, ^234^, ^260^ ^]^ In addressing this issue, Bard put forward the concept of the Z‐scheme heterojunction (direct/indirect (electron mediator‐assisted) Z‐scheme heterojunction) with a maximized redox potential.^[^ ^267^ ^]^ For the indirect Z‐scheme heterojunction, under the light excitation, the worthless electrons that generated at the low‐potential semiconductor (PSII) transferred and reacted with the holes on the high‐potential semiconductor (PSI) through the electron mediator (Pt and Au, Figure 9D).^[^ ^110^, ^234^, ^260^ ^]^ The remaining and effective holes on the PSII and the electrons on the PSI consume their accumulation through redox reactions, thereby achieving efficient separation of light‐generated charges. For instance, Li et al. designed and prepared a novel ternary Z‐scheme PCN/silver (Ag)/silver phosphate (Ag_3_PO_4_) photocatalyst using a two‐step method (Figure 10E).^[^ ^229^ ^]^ The optimal PCN/Ag/Ag_3_PO_4_ shows a 74% removal rate for NO*_x_*, which was higher than that of the sole PCN and Ag_3_PO_4_. The superior performance was attributed to the improved thermodynamics and kinetics, which endowed promoted mass transfer, charge separation and transmission, optical capability, and physical/chemical properties. In addition, other nonmetal electron‐medicated indirect Z‐scheme heterojunction is also developed to improve the NO*_x_* abatement efficiency driven by visible light. For instance, Wang et al. successfully prepared unique layered iodine (I)‐doped BiOIO_3_/PCN heterojunction by the method of exchange‐solvothermal (Figure 10F).^[^ ^228^ ^]^ The strong PODN activity of BiOIO_3_/I^–^/PCN at the ppb‐level was endowed by the close interfacial contact of the Z‐scheme heterojunction, namely, the promoted charge transport efficiency and wide optical range that improved the PODN efficiency was caused by the synergistic heterojunction.

Different from the indirect Z‐scheme heterojunction, the charge transfer process of the direct Z‐scheme heterojunction does not need any electronic transmission mediator, and it can also form a built‐in electric field spatially to optimize the redox potential of the semiconductors.^[^ ^110^, ^234^, ^260^ ^]^ This type of charge transfer makes the preparation process of the indirect Z‐scheme heterojunction simpler than that of the direct Z‐scheme heterojunction. For example, Dong et al. constructed a direct Z‐scheme heterojunction consisting of PCN and perylene imides (PI) to address the issues (incomplete oxidation of NO and the photocatalyst deactivation).^[^ ^184^ ^]^ The Z‐scheme PI/PCN heterojunction exhibited significant PODN activity (compared with pristine PCN, Figure 10G). The improved PODN activity was attributed to the efficient charge separation of Z‐scheme PI/PCN heterojunctions, which provided stronger oxidized power for the holes and reduced power for electrons, thereby accelerating the ROS generation and NO conversion. The construction of the Z‐type heterojunction not only solves the problem that the photogenerated charge recombines easily in the single‐component semiconductor but also solves the problem of the reduced redox ability of the semiconductors due to the imperfect charge transfer pathway in the traditional heterojunction.

Recently, a novel S‐scheme heterojunction is proposed to address the shortcomings of conventional type‐II heterojunction by Fu et al.^[^ ^268^ ^]^ The S‐type heterojunction is mainly composed of two n‐type semiconductors (reducing photocatalyst (RP) and oxidizing photocatalyst (OP)). Its band structure is similar to the traditional type II heterojunction, but the type of charge transfer is different. Due to the difference in work function and Fermi level between OP and RP, a built‐in electric field in space is formed. When the two n‐type semiconductors are in contact, their Fermi levels will be rearranged and cause their energy bands to bend. It will promote the recombination of meaningless light‐excited electrons and holes; and effective electrons and holes are retained to participate in following redox reactions, thereby encouraging the efficient separation of photogenerated carriers. For instance, Ren et al. fabricated a novel S‐scheme Sb_2_WO_6_/PCN nanocomposite by an ultrasound‐assisted method.^[^ ^230^ ^]^ The S‐scheme Sb_2_WO_6_/PCN nanocomposite exhibited excellent performance for PODN activity driven by visible light because of the boosted charge dynamics and improved ROS generation (Figure 10H). Therefore, PCN‐based heterojunctions have indeed made great achievements in PODN reaction; however, it is still in its infancy for practical application and commercialization.

### 3.2 PCN for Desulfurization

The emission of sulfur compounds accelerates the erosion of the historical building, destroys soil chemistry, and disturbs the pH of water bodies, thereby jeopardizing marine life.^[^ ^10^, ^11^, ^12^, ^269^ ^]^ Moreover, breathing in a toxic climate can result in numerous health concerns (e.g., asthma, heart disease, and respiratory syndrome).^[^ ^11^, ^270^, ^271^, ^272^ ^]^ Notably, the source of sulfur compounds emissions is primarily produced by the random and massive release of sulfur fuel oil combustion waste, subsequently causing air pollution, health concerns, acid rain, and global warming.^[^ ^11^, ^12^, ^272^, ^274^ ^]^ Considering the above‐mentioned negative and harmful influences of sulfide and the needs of social development, exploring an efficient desulfurization strategy to degrade the sulfur hazards and inhibit sulfide production is necessary.

**Figure 11:**
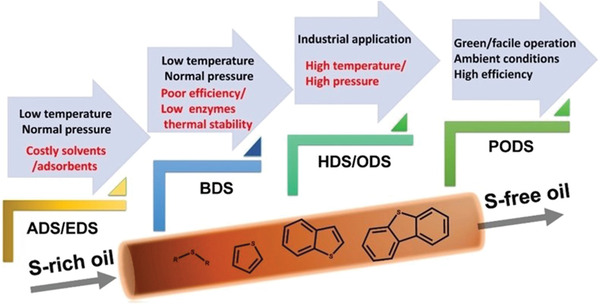
General features and development course of the removal of sulfur compounds. Reproduced with permission.^[^ ^12^ ^]^ Copyright 2021, Wiley‐VCH.

Sulfur‐removal technologies can be generally characterized into two categories: hydrodesulfurization (HDS) technology and non‐HDS technology (**Figure** **11**).^[^ ^11^, ^12^, ^275^, ^276^, ^277^ ^]^ HDS is widely adapted and revised the desulfurization process in the industry. It can produce low‐sulfur oil; however, the toxic hydrogen sulfide and the reduced octane number in fuel oil are also produced. In addition, HDS is operated under harsh operating conditions, including reacting with H_2_ at high temperatures (200–450 °C) and pressures (3–6 MPa), and employing expensive catalysts, causing the inability of HDS to produce superlative desulfurization efficiencies.^[^ ^278^, ^279^, ^280^, ^281^, ^282^ ^]^ Given the limited efficiency and high cost of HDS, non‐HDS technology as an advancement of effective alternative deep desulfurization technologies has been considered, including physical desulfurization (PDS), biological desulfurization (BDS), and chemical desulfurization (CDS).^[^ ^11^, ^12^, ^275^, ^276^, ^277^, ^278^, ^283^, ^284^, ^285^ ^]^ PDS can be performed under mild conditions (low temperature and pressure); however, the adsorption capacity and adsorbent regeneration are rather limited; and it is confronted with a complex and high‐cost recovery of the extraction agent process.^[^ ^278^, ^286^ ^]^ The development of BDS still faces extreme problems regarding its practical application because of its requirement and limitation of complex reaction equipment/technology, high desulfurization cost, and inefficient biocatalyst.^[^ ^12^, ^284^ ^]^ The CDS technology exhibits the merits of high desulfurization efficiency and robust catalytic stability, but the high cost, large loss of catalyst, and secondary environmental pollution are identified during the desulfurization process.^[^ ^285^, ^287^ ^]^ Therefore, developing more efficient desulfurization technologies is necessary, which promotes the deep oxidation of the desulfurization process while reduces the process cost and energy consumption.

Photocatalytic oxidation desulfurization (PODS) is essentially an advanced version of ODS, referring to efficient ROS generation and then react with the C–S–C bond of sulfur compounds, which has received considerable research interest because of its economic, sustainable, and reusable advantages.^[^ ^11^, ^12^, ^104^, ^288^, ^289^ ^]^ Under this process, sulfur compounds (e.g., BT: benzothiophene, RSH: *n*‐dodecanethiol, 4,6‐DMDBT: 4,6‐dimethyl dibenzothiophene, Th: thiophene, DBT, CH_3_SH, etc.) can be converted to SO_4_^2–^, or matching sulfone, and then be absorbed and extracted by an alkaline solution/polar solvent.^[^ ^290^, ^291^, ^292^, ^293^ ^]^ To date, a significant development with kinds of photocatalysts is achieved for PODS reaction, which is primarily for inorganic substances (e.g., CeO_2_, Fe_2_O_3_, TiO_2_, and WO_3_) to metal‐free organic polymer (PCN).^[^ ^107^, ^293^, ^294^, ^295^, ^296^ ^]^ The PCN comprised of sp^2^‐hybridized carbon and nitrogen atoms has been considered as a suitable species with a particular electronic structure, good physicochemical stability, and facile synthesis.^[^ ^45^, ^49^, ^82^, ^297^ ^]^ For instance, in 2014, the TiO_2_/PCN composites that employed in photocatalytic oxidative desulfurization received a surprising activity, which was ascribed to the increased absorption of the optical region.^[^ ^293^ ^]^ This work implied the greatest potential of PCN utilization in PODS applications, attracting considerable attention from researchers. However, the PODS efficiency remains moderate because of the low specific surface area of the PCN. In 2015, Zhu et al. used PCN with enlarged surface area as the support of heteropoly acid, and the obtained catalyst displayed excellent reusability in the ODS process.^[^ ^298^ ^]^ In 2016, MPCN as a single heterogeneous photocatalyst was first introduced into the ODS reaction, which could oxidate DBT with visible light and reached 100% under optimal conditions for the desulfurization rate of model fuel.^[^ ^107^ ^]^ Given the suitable band position of the PCN, the photogenerated electrons of CB that were excited by the visible light exhibited an extraordinary capacity to reduce O_2_ and formed a strong oxidized O_2_^⋅–^. The intermediate products are mineralized and converted to SO_4_^2–^ and the corresponding sulfone after combining with the ROS or h^+^, and the specific conversion processes are shown in the following equations^[^ ^11^ ^]^

(35) $$\mathrm{PCN}+h\nu \to e^{-}+h^{+}$$

(36) $$O_{2}+e^{-}\to {O_{2}}^{\cdot -}$$

(37) $$O_{2}\cdot -+2H^{+}+e^{-}\to H_{2}O_{2}$$

(38) $$H_{2}O_{2}+e^{-}\to \cdot \mathrm{OH}+OH^{-}$$

(39) $${O_{2}}^{\cdot -}+2H^{+}+2e^{-}\to \cdot \mathrm{OH}+OH^{-}$$

(40) $$2\mathrm{DBT}+{O_{2}}^{\cdot -}\to 2\mathrm{DBTO}$$

(41) $$2\mathrm{DBT}+{O_{2}}^{\cdot -}\to 2\mathrm{DBT}O_{2}$$

(42) $$\cdot \mathrm{OH}+\mathrm{DBT}\to \mathrm{DBT}O_{2}+H^{+}$$

However, PCN still has limitations, which needs to be addressed, including insufficient optical absorption, inhibited charge dynamics, inadequate reaction sites, and agglomerate photocatalysts.^[^ ^45^, ^49^, ^82^, ^299^ ^]^ In addressing the above‐mentioned obstacles, nanostructure design, electronic structure modulation, and heterostructure construction based on PCN are used to achieve efficient PODS activity.^[^ ^107^, ^116^, ^130^ ^]^ Considering the limited amount of PODS reviews, we hypothesize that summarizing the recent development of PCN on PODS reaction is necessary. Some recently reported PCN‐based photocatalysts with various strategies used in POND are shown in **Table** **2**.

**Table 2:** Summary of recently reported PCN‐based photocatalysts utilized in desulfurization reaction

Photocatalysts	Types	Experimental conditions	Light source	Analyzer	Main products and sulfur compounds removal ratio	Refs.
Mesoporous PCN	Nanostructure design	Reactor: 100 mL Cat.^a)^: 0.1 g Conc.^b)^: 100 ppmw Time^c)^: 100 min S Source: DBT^d)^, 4,6‐DMDBT^e)^, and BT^f)^ Solvent: *n*‐octane	300 W Xe lamp (*λ* > 420 nm)	WK‐2D	Products: DBTO_2_ *η* ^g)^ (S): 100% (4,6‐DMDBT: 30 min.; DBT and BT: 90 min)	^[^ ^107^ ^]^
Na/PCN	Electronic structure regulation	Reactor: 20 mL Cat.: 0.1 g Conc.: 200 µg g^−1^ Time: 360 min S Source: DBT Solvent: *n*‐octane	300 W Xe lamp (*λ* > 420 nm)	Agilent GC^h)^‐7890B	Products: SO_4_^2–^‐ *η*(S): 66.1%	^[^ ^300^ ^]^
Ag/PCN	Electronic structure regulation	Reactor: unmarked Cat.: 0.1 g Conc.: 5 ppm Time: 180 min S Source: CH_3_SH Solvent: no	W‐type fluorescent lamps (*λ* > 420 nm)	GC‐14B with FID^i)^	Products: SO_4_^2–^‐ *η*(S): 93%	^[^ ^116^ ^]^
Ti_3_C_2_/PCN	Heterostructure construction	Reactor: 20 mL Cat.: 0.05 g Conc.:140 µg g^−1^ Time: 180 min S Source: Th^j)^ Solvent: *n*‐octane	300 W Xe lamp (*λ* > 420 nm)	Agilent GC‐7890B	Products: SO_4_^2–^‐ *η*(S): 72%	^[^ ^66^ ^]^
CeF_3_/PCN	Heterostructure construction	Reactor: 200 mL Cat.: 0.1 g Conc.:140 µg g^−1^ Time: 180 min S Source: DBT Solvent: *n*‐octane	300 W Xe lamp (*λ* > 420 nm)	UV fluorescence	Products: DBTO_2_‐ *η*(S): 84.2%	^[^ ^301^ ^]^
(Bmin)_3_PMo_12_O_40_/PCN	Heterostructure construction	Reactor: 25 mL Cat.: 0.05 g Conc.:1000 ppm Time: 240 min S Source: DBT Solvent: *n*‐octane	250 W high‐pressure sodium lamp	Agilent GC‐6890	Products: DBTO_2_ *η*(S):99.1% (DBT)	^[^ ^302^ ^]^
ZnTcPc/PCN	Heterostructure construction	Reactor: 100 mL Cat.: 0.02 g Conc.: 800 *μ*L L^–1^ Time: 240 min S Source: Th Solvent: *n*‐octane	400 W halogen lamp	Agilent GC‐6890	Products: SO_4_^2–^‐ *η*(S): 72%	^[^ ^303^ ^]^
TiO_2_/PCN	Heterostructure construction	Reactor: 20 mL Cat.: 0.2 g Conc.: 500/250 ppm Time: 120 min S Source: DBT, BT and RSH^k)^ Solvent: acetonitrile	250 W high pressure Hg lamp (UV light)	Agilent GC‐7890A	Products: BTO_2/_DBTO_2_ *η*(S): 100% (DBT); ≈90% (BT/RSH)	^[^ ^293^ ^]^
CeO_2_/ATP^l)^/PCN	Heterostructure construction	Reactor: 200 mL Cat.: 0.1 g Conc.:200 ppm Time: 180 min S Source: DBT Solvent: *n*‐octane	300 W Xe lamp (*λ* > 420 nm)	UV fluorescence (THA2000S)	Products: DBTO_2_ *η*(S): 98%	^[^ ^129^ ^]^
BiVO_4_/PCN/SiO_2_	Heterostructure construction	Reactor: unmarked Cat.: 0.2 g Conc.: 300 ppm Time: 300 min S Source: DBT Solvent: dodecane	Xe lamp (*λ* > 420 nm)	GC/FID‐9790^m)^	Products: DBTO_2_ *η*(S): 99%	^[^ ^119^ ^]^
CuO–ZnO/PCN	Heterostructure construction	Reactor: 120 mL Cat.: 0.05 g Conc.: 150 ppm Time: 30 min S Source: DBT Solvent: ethanol	200 W lamp (*λ* > 420 nm)	Agilent GC 7890/MS^n)^−5975C	Products: DBTO_2_ *η*(S): 84.5%	^[^ ^304^ ^]^
ZnM‐LDHs/PCN	Heterostructure construction	Reactor: 250 mL Cat.: 0.18 g Conc.: 150 ppm Time: 180 min S Source: DBT, 4,6‐DMDBT, and BT Solvent: acetonitrile	500 W mercury lamp (*λ* > 420 nm)	KY3000‐SN	Products: DBTO_2_ *η*(S): 96.8%	^[^ ^305^ ^]^
BiOI/I^3–^/PCN	Heterostructure construction	Reactor: 120 mL Cat.: 0.05 g Conc.: 70 ppm Time: 30 min S Source: CH_3_SH Solvent: ethanol	8 W LED belt (440–490 nm)	Agilent GC‐7890A	Products: SO_4_^2–^ *η*(S): 94.17%	^[^ ^130^ ^]^

^a)^ The mass of the photocatalysts (Cat.)

^b)^ The concentration of Sulfur compounds (Conc.)

^c)^ Irradiation time (Time)

^d)^ Dibenzothiophene (DBT)

^e)^ 4,6‐Dimethyl dibenzothiophene (4,6‐DMDBT)

^f)^ Benzothiophene (BT)

^g)^ The removal efficiency *η* (%) of pollutant was calculated as: *η* (%) = (1 − *C*/*C*_0_) × 100%

^h)^ Gas chromatograph (GC)

^i)^ Flame ionized detector (FID)

^j)^ Thiophene (Th)

^k)^ *n*‐Dodecanethiol (RSH)

^l)^ Attapulgite (ATP)

^m)^ GC/FID‐9790, Fuli, Hangzhou, China (GC/FID‐9790)

^n)^ Mass spectrometer (MS).

#### 3.2.1 Research Progress of PCN for Desulfurization

##### Regulating Molecular Structure

In general, heteroatom doping is considered to be an effective and recommendable strategy to alter the electronic structures of the PCN.^[^ ^49^, ^82^, ^84^, ^245^ ^]^ This modification will endow PCN with unoccupied molecular orbitals. This promotes the charge separation capability, hindering the recombination of solar‐excited electron–hole pairs, and contributes toward visible‐light responsivity. For instance, Zhang et al. reported the NaCl‐assisted uniform doping of Na in PCN matrix via a method of mixed‐calcination.^[^ ^300^ ^]^ The synthetic method resulted in the production of a porous and highly dispersed structure, thereby improving the surface energy, increasing the optical‐harvesting capability, and retarding the recombination of solar‐excited electron–hole pairs. During the whole PODS process, a promotion effect was produced by ROS generation from the reaction of electron and O_2_. Consequently, the as‐synthesized photocatalysts revealed excellent Th desulfurization performance.

##### Constructing Composites

MXenes as layered materials have been reported and participated in various fields.^[^ ^306^, ^307^ ^]^ A composition of semiconductors and MXene may be used to produce a Schottky heterostructure to enhance the performance of photocatalytic reaction by primarily facilitating the dynamics of photoinduced electrons and holes.^[^ ^66^, ^308^, ^309^ ^]^ A Ti_3_C_2_ (typical MXene) as an efficient and low‐cost co‐catalyst was chosen to couple with PCN and then applied in the PODS reaction by Li et al.^[^ ^66^ ^]^ Constructing a Schottky barrier composed of Ti_3_C_2_ and PCN could effectively improve the kinetic efficiency of photogenerated charges, provide an ideal electronic storage library, and promote the generation of ROS to accelerate the transformation of the final product. In addition, PCN nanosheets that dispersed on mesoporous silica and then hybridized with visible‐light‐responsive BiVO_4_ exhibited as high as 99% conversion of DBT.

**Figure 12:**
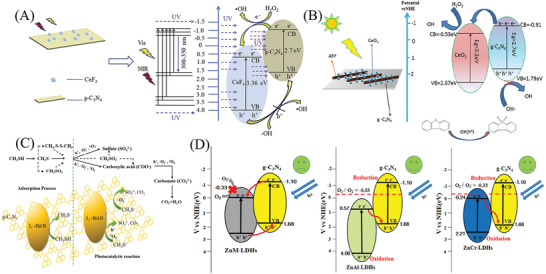
A) Schematic illustration of the photocatalytic mechanism of the CeF_3_/PCN composite. Reproduced with permission.^[^ ^301^ ^]^ Copyright 2020, Elsevier. B) Photocatalytic desulfurization mechanism of CeO_2_/ATP/PCN ternary nanocomposite. Reproduced with permission.^[^ ^129^ ^]^ Copyright 2017, Elsevier. C) Conversation pathway of adsorption and photocatalytic oxidation of CH_3_SH. Reproduced with permission.^[^ ^130^ ^]^ Copyright 2018, American Chemical Society. D) Z‐scheme heterostructure of the photocatalytic oxidation of ZnM‐LDHs/PCN (M = Al, Cr). Reproduced with permission.^[^ ^305^ ^]^ Copyright 2020, American Chemical Society.

Heterojunction photocatalysts construction based on coupling two semiconductors depend on the band potential of the CB/VB and band alignment from a thermodynamic point of view.^[^ ^49^, ^82^, ^109^, ^256^, ^261^ ^]^ In addition, heterojunction photocatalysts can solve the drawbacks and limits of single‐component catalysts endowed with weak redox capability, inefficient catalytic efficiency, and inappropriate CB and VB positions. The CeF_3_ photocatalyst has aroused much attention and is regarded as an optical component because of the contribution of charge, optical, and ionic conductivity response.^[^ ^310^ ^]^ The introduction of CeF_3_ into the PCN by constructing a heterojunction is viewed as a potential photocatalyst to increase the activity and stability. For instance, Lu et al. utilized CeF_3_ nanoparticles to decorate the PCN nanosheets and fabricate CeF_3_/PCN nanocomposites with well band alignment by the method of microwave hydrothermal (Figure 12A).^[^ ^312^ ^]^ The well‐defined CeF_3_/PCN heterojunction could be endowed with the effective separation of photogenerated electrons and holes, and the synergistic effect of the CeF_3_/PCN type II nanocomposite exhibited the superior degradation rate of DBT with visible light. Similar and positive results could be achieved from type II zinc phthalocyanine/PCN (ZnTcPc/PCN) composites by Zhang et al.^[^ ^303^ ^]^ In addition, the ternary CeO_2_/attapulgite (ATP)/PCN noncompounds were created by a facile self‐assembly method, which effectively produced an enlarged surface area (enhanced adsorption and reaction sites) and prevented the generation of restacked PCN.^[^ ^129^ ^]^ The extended adsorption edge of the optical and the facilitated kinetics of charges was also achieved by the synergetic effect of CeO_2_/PCN type II heterojunctions, thereby converting 98% of DBT and exhibiting excellent recyclability (**Figure** **12**B).

Different from the type II heterojunctions, the Z‐scheme heterojunctions maximize the redox ability of the semiconductor component through a zigzag charge transfer mechanism and provide an IEF to promote the separation/transmission dynamics of the electron–hole pairs.^[^ ^82^, ^110^, ^255^, ^260^ ^]^ For instance, Hu et al. synthesized a self‐stabilized Z‐scheme heterojunction photocatalyst of I^3–^‐containing BiOI ultrathin nanosheets/porous PCN (BiOI/I^3–^/PCN) by a facile solvothermal method (Figure 12C).^[^ ^130^ ^]^ The indirect BiOI/I^3–^/PCN heterojunction presented a dramatically PODS efficiency toward CH_3_SH removal in comparison with pure BiOI and PCN because of the promoted separation dynamics and redox potential of light‐excited charge. The pathway of photocatalytic CH_3_SH removal suggested the partial oxidization of the adsorbed CH_3_SH and the cleaved C–S bond, which was primarily endowed by the active N atoms (PCN) and Bi‐metal (BiOI), thereby facilitating the formation and oxidation capacity of ROS.

Huang et al. successfully synthesized direct Z‐scheme photocatalysts of ZnM‐LDHs/PCN (M = Al, Cr) by electrostatic self‐assembly (Figure 12D).^[^ ^305^ ^]^ Photocatalytic desulfurization of ZnAl‐LDHs/PCN and ZnCr‐LDHs/PCN could reach above 95%, which was primarily ascribed to the improved carrier transfer, the suppressed electron–hole recombination, and the improved redox potential. A triplex CuO–ZnO/PCN dual Z‐scheme heterojunction was achieved via a multistep ultrasound‐assisted hydrothermal procedure, showing a 99.1% desulfurization rate and excellent stability under visible‐light‐irradiation.^[^ ^304^ ^]^ The outstanding photocatalytic performance was originated from the improved specific surface area and charge kinetics favored by the production of dual Z‐scheme CuO‐ZnO/PCN nanocomposites.

### 3.3 PCN for VOC Removal

VOC (formaldehyde (HCHO), benzene, toluene, ethylbenzene, and xylene (BTEX)) are primarily produced by exhaust gas from fuel combustion and chemicals from indoor decoration materials, which are ubiquitous in the atmospheric environment, posing great harm to human health.^[^ ^13^, ^14^, ^15^, ^16^, ^311^, ^312^, ^313^ ^]^ A variety of approaches are identified for controlling VOC in indoor environments such as source control, ventilation, and air cleaning.^[^ ^314^, ^315^, ^316^, ^317^, ^318^ ^]^ Large equipment (e.g., a light source, plasma equipment, or heating equipment) is necessary for the emission source control and ventilation, making this process complicated and increasing footprint costs.^[^ ^132^, ^319^, ^320^ ^]^ At present, air purification gradually remains to be a potential method because of the restraint in the secondary environmental pollution production, which involves diverse control techniques, for example, physical adsorption, biological treatment and chemical oxidation.^[^ ^132^, ^314^, ^315^, ^316^, ^317^, ^318^, ^321^ ^]^ Compared with physical adsorption and biological treatment, the photocatalytic decomposition of VOC is considered to be a potential and sustainable chemical oxidation method due to the contribution of its high efficiency and reusability, simple operation, and nonpolluting characteristics.^[^ ^322^, ^323^ ^]^ It can degrade VOC to low‐toxicity products (carbon dioxide (CO_2_) and water (H_2_O)) without generating other harmful by‐products at ambient temperature, which promotes long‐term environmental protection.^[^ ^324^, ^325^ ^]^

In the past decades, several kinds of research have been conducted and investigated in the photocatalytic decomposition of VOC, which address indoor pollution issues.^[^ ^322^, ^323^ ^]^ TiO_2_ as a dominant photocatalyst is extensively studied and carried out in the VOC decomposition because of the advantages of the suitable oxidative capability, superior anticorrosion and reusability.^[^ ^326^ ^]^ However, the response of the TiO_2_ to UV light (≈4% of solar energy) seriously limits its practical application. The metal‐free and visible‐light‐responsive PCN catalysts have gained increasing interest, which is ascribed to the enlarged specific surface area for mass transfer and reactive sites supply, suitable and adjustable electronic structure for ROS generation, abundant functional groups/defects for coupling other useful species.^[^ ^45^, ^49^, ^82^ ^]^ These characteristics lead to boosted chemical stability, optical absorption, and adsorption capacity for the gas molecules, showing great potential for photocatalytic VOC removal. For the first time, Yu et al. created a direct TiO_2_/PCN Z‐scheme noncompounds through a facile calcination route.^[^ ^106^ ^]^ The introduced PCN‐based Z‐scheme noncompounds showed a great influence on the photocatalytic decomposition of HCHO in the air because of the efficient spatial separation kinetics of light‐induced charge carriers. In general, the formation processes of the intermediates during the photooxidation of HCHO are shown in the following equations^[^ ^138^ ^]^

(43) $$\mathrm{PCN}+h\nu \to e^{-}+h^{+}$$

(44) $$O_{2}+e^{-}\to {O_{2}}^{\cdot -}$$

(45) $$h^{+}+H_{2}O\to \cdot \mathrm{OH}+H^{+}$$

(46) $$\mathrm{HCHO}+\cdot \mathrm{OH}\to \cdot \mathrm{CHO}+H_{2}O\overset{\cdot \mathrm{OH}}{\to }\cdot \mathrm{OHHCOOH}$$

(47) $$\cdot \mathrm{CHO}+{O_{2}}^{\cdot -}\to \mathrm{HC}{O_{3}}^{-}\overset{H^{+}/\mathrm{HCHO}}{\to }H^{+}/\mathrm{HCHOHCOOH}$$

(48) $$\mathrm{HCOOH}+\cdot \mathrm{OH}/h^{+}\to CO_{2}+H_{2}O/CO_{2}+H^{+}$$

However, the efficiency of PCN photocatalysts on removing VOC is still moderate because of the limited utilization of the visible light, sluggish charge kinetics and severe aggregations of photocatalysts. The electronic structure regulation and heterostructure construction on PCN are rapidly developed to enlarge the exploitation of PCN photocatalysts on VOC removal, showing the importance and development of this field.^[^ ^63^, ^106^, ^118^, ^135^ ^]^ Here, we reviewed the catalyst preparation/synthesis, measurements of catalytic activity, and discussion/understanding of the activity enhancement and catalytic mechanism in the VOC photooxidation. Some recently reported PCN‐based photocatalysts with various strategies used in VOC removal are shown in **Table** **3**.

**Table 3:** Summary of recently reported PCN‐based photocatalysts utilized in VOC removal

Photocatalysts	Types	Experimental conditions	Light source	Analyzer	Main products and VOC removal ratio	Refs.
Porous PCN nanosheets	Nanostructure design	Reactor: 1.5 L Cat.^a)^: 0.5 g Conc.^b)^: 20 L VOC: HCHO Time^c)^: 6 h	Yellow LED light (*λ* = 585 nm)	Spectrophotometer	Products: CO_2_, H_2_O *η* ^d)^(HCHO): 56.32%	^[^ ^327^ ^]^
K/PCN	Electronic structure regulation	Reactor: 6 L Cat.: 0.1 g Conc.: 300 ppm VOC: HCHO Time: 24 min	350 W Xe lamp (*λ* > 420 nm)	Innova 1412^e)^	Products: CO_2_, H_2_O *η*(HCHO): 90%	^[^ ^118^ ^]^
Ag/PCN	Electronic structure regulation	Reactor: 1 L Cat.: 4 mg Conc.: 700 ppmv VOC: toluene Time: 6–8 h	6 W LED lamps (*λ* = 400 nm)	Agilent GC 6890	Products: CO_2_, H_2_O *η*(HCHO): 95%	^[^ ^328^ ^]^
Sb/PCN	Electronic structure regulation	Reactor: 6 L Cat.: 0.4 mg cm^−2^ Conc.: 1 L VOC: acetone, styrene, and cumene Time: 180 min	6 W daylight lamps (*λ* > 390 nm)	GC‐2010 Plus	Products: CO_2_, H_2_O *η*(acetone/ cumene): ≈80%	^[^ ^329^ ^]^
C/PCN	Electronic structure regulation	Reactor: 20 L Cat.: 1 g Conc.: 50 ppm VOC: HCHO Time: 300 min	250 W HPML^f)^ (*λ* > 420 nm)	TU‐1901^g)^	Products: CO_2_, H_2_O *η*(HCHO): 84.63%	^[^ ^325^ ^]^
C/O co‐doped PCN	Composite construction	Reactor: 20 L Cat.: 50 mg Conc.: 1 mL VOC: toluene Time: 180 min	300 W Xe lamp (*λ* > 420 nm)	GC‐2010	Products: BZH *η*(HCHO): 1.02%	^[^ ^330^ ^]^
Bi_12_TiO_20_ /PCN	Composite construction	Reactor: 600 mL Cat.: 0.5 g Conc.:160 ppm VOC: HCHO Time: 420 min	300 W Dy lamp (*λ* ≥ 400 nm)	Innova 1412	Products: CO_2_ *η*(HCHO): 75%	^[^ ^331^ ^]^
Ag_3_PO_4_/PCN	Composite construction	Reactor: 42 mL Cat.: 0.05 g Conc.: 0.5 mg m^–3^ VOC: HCHO Time: 600 min.	420 nm monochrome LED	POV‐18^h)^	Products: CO_2_ *η*(HCHO): 22.4%	^[^ ^332^ ^]^
Ag‐ZnO/PCN	Composite construction	Reactor: 40 L Cat.: 0.2 g Conc.:1.7 ppm VOC: HCHO Time: 180 min	350 W Xe lamp	GAS Tiger 2000‐CH_2_O‐L^i)^	Products: CO_2_ *η*(HCHO): 81.2%	^[^ ^138^ ^]^
TiO_2_/PCN/waste zeolites	Composite construction	Reactor: 40 L Cat.: 0.15 g Conc.:1.2 ppm VOC: HCHO Time: 180 min	300 W Xe lamp (*λ* >420 nm)	C‐XP‐308B^j)^	Products: CO_2_ *η*(HCHO): 81.2%	^[^ ^333^ ^]^
Bi_2_MoO_6_/Bi/PCN	Composite construction	Reactor: 2.2 L Cat.: 0.015 g Conc.:1600 ppm VOC: HCHO Time: 600 min	300 W Xe lamp (*λ* > 420 nm)	Online gas infrared detector and GC^k)^ detector	Products: CO_2_, H_2_O *η*(HCHO): 98.80%	^[^ ^135^ ^]^
AgFeO_2_/PCN	Composite construction	Reactor: 100 mL Cat.: 0.5 g Conc.: 20 mg L^−1^ VOC: HCHO Time: 180 min	300 W Dy lamp (*λ* > 400 nm)	UV1750^l)^	Products: CO_2_ *η*(HCHO): 94%	^[^ ^334^ ^]^
TiO_2_/PCN	Composite construction	Reactor: 15 L Cat.: 0.3 g Conc.: 170 ppm VOC: HCHO Time: 50 min	365 nm UV lamp	Model UV‐A^m)^	Products: CO_2_, H_2_O *η*(HCHO): ≈90%	^[^ ^106^ ^]^
STO/TN/N‐PCN	Composite construction	Reactor: unmarked Cat.: 0.2 g Conc.: 1000 ppm VOC: toluene Time: 360 min	300W Xe lamp (*λ* > 420 nm)	GC	Products: CO_2_ *η*(toluene): ≈93%	^[^ ^335^ ^]^
BiVO_4_/PCN	Composite construction	Reactor: unmarked Cat.: 0.1 g Conc.: 25 ppm VOC: toluene Time: 480 min	300 W Xe lamp (*λ* > 400 nm)	GC/FID 9790^n)^	Products: CO_2_ *η*(toluene): 68.2%	^[^ ^336^ ^]^
WO_3_/PCN	Composite construction	Reactor: unmarked Cat.: unmarked Conc.: 2000 ppm VOC: toluene Time: 48 h	LED lamp (435 nm)	GC‐8A^o)^	Products: CO_2_ *η*(toluene): 80%	^[^ ^63^ ^]^
PCN nanosheets/textile	Nanostructure design	Reactor: 10 L Cat.^a)^: 0.5 g Conc.^b)^: 100 ppm VOC: HCHO Time^c)^: 20 min	300 W Xe lamp (*λ* > 420 nm)	GC‐2014C	Products: CO_2_, H_2_O *η* ^e)^(HCHO): 100%	^[^ ^337^ ^]^
Ag‐BTiO_2_/PCN	Composite construction	Reactor: 83.2 mL Cat.: 0.5 g Conc.: 1 ppm VOC: *m*‐xylene Time: 300 min	8 W lamp (*λ* = 400‒720 nm)	GC‐MS	Products: CO_2_ *η*(*m*‐xylene): 79%	^[^ ^338^ ^]^
H3PW12O40/PCN	Composite construction	Reactor: 300 mL Cat.: 135 mg Conc.: 500 L VOC: *m*‐xylene Time: 120 min	300 W Xe lamps	Agilent 1200 HPLC	Products: BZH, CO_2_ *η*(*m*‐xylene): 90.1%	^[^ ^313^ ^]^
V_2_O_5_/PCN	Composite construction	Reactor: 53 mL Cat.: 30 mg Conc.: 0.1 ppm VOC: *p*‐xylene Time: unmarked	150 W Xe lamp	GC‐MS	Products: CO_2_ *η*(*p*‐xylene): 97%	^[^ ^339^ ^]^
Bi_2_MoO_6_/PCN	Composite construction	Reactor: 25 mL Cat.: 40 mg Conc.: 0.1 ppm VOC: toluene Time: 180 min	300 W Xe lamp (*λ* > 400 nm)	GCMS‐QP2010	Products: BZH *η*(toluene): ≈90%	^[^ ^340^ ^]^
MnO*_x_*/PCN	Composite construction	Reactor: unmarked Cat.: 0.4 mg cm^–2^ Conc.: 700 ppmv VOC: toluene Time: 6–10 h	6 W sunlight‐type lamps	Agilent GC 6890	Products: BZH, CO_2_ *η*(toluene): ≈85%	^[^ ^341^ ^]^
TiO_2_/PCN	Composite construction	Reactor: unmarked Cat.: 0.4 mg cm^−2^ Conc.: 100–200 ppmv VOC: toluene Time: 4 h	6 W Fluorescent UV	Agilent GC 6890	Products: BZH, CO_2_ *η*(toluene): 96.4%	^[^ ^342^ ^]^
CeO_2_–TiO_2_/PCN	Composite construction	Reactor: unmarked Cat.: 40 mg Conc.: 700 ppmv VOC: toluene Time: 6–10 h	6 W Fluorescent UV	Agilent GC 6890	Products: BZH, CO_2_ *η*(toluene): 95%	^[^ ^343^ ^]^
ZnO/NiMoO_4_/PCN	Composite construction	Reactor: 1.9 L Cat.: unmarked Conc.: 4000 *μ*mol L^–1^ VOC: toluene Time: 120 min	8 W ultraviolet lamp	Agilent 7890A	Products: BZH, CO_2_ *η*(toluene): 95%	^[^ ^344^ ^]^
BiPO_4_/PCN	Composite construction	Reactor: 120 mL Cat.: 0.1 g Conc.: 120 ppm VOC: toluene Time: 240 min	500 W Xe lamp (*λ* > 400 nm)	Agilent GC 7890A	Products: BZH, CO_2_ *η*(toluene): 82%	^[^ ^345^ ^]^
In_2_S_3_/PCN	Composite construction	Reactor: unmarked Cat.: 50 mg Conc.: unmarked VOC: toluene Time: 180 min	500 W Xe lamp (*λ* >400 nm)	GC‐MS	Products: BZH, CO_2_ *η*(toluene): 89.7%	^[^ ^346^ ^]^
ZSM‐4/Mn‐PCN	Composite construction	Reactor: unmarked Cat.: 1 g Conc.: 25 ppm VOC: toluene Time: 90 min	4 W VUV lamps	GC‐MS	Products: BZH, CO_2_ *η*(toluene): 90%	^[^ ^347^ ^]^
TiO_2_/PCN/cotton fabrics	Composite construction	Reactor: 350 mL Cat.: 0.58 g Conc.: 0.2 µL VOC: toluene Time: 50 min	4 W VUV lamps	GC‐MS	Products: BZH, CO_2_ *η*(toluene): 93.64%	^[^ ^348^ ^]^
BiVO_4_/PCN	Composite construction	Reactor: 10 mL Cat.: 100 mg Conc.: 25 ppm VOC: toluene Time: 8 h	300 W Xe lamps (*λ* > 400 nm)	GC/FID‐9790	Products: BZH, CO_2_ *η*(toluene): 68.2%	^[^ ^336^ ^]^
Cs*_x_* WO_3_/PCN	Composite construction	Reactor: 10 mL Cat.: 50 mg Conc.: 200 ppm VOC: HCHO Time: 240 min	300 W Xe lamps (*λ* > 400 nm)	Model 1412	Products: BZH, CO_2_ *η*(HCHO): 100%	^[^ ^312^ ^]^
NiWO_4_/PCN	Composite construction	Reactor: 1 L Cat.: 0.5 g Conc.: 1000 ppm VOC: toluene Time: 240 min	100 W lamps (*λ* > 400 nm)	GC‐FID	Products: BZH, CO_2_ *η*(toluene): 100%	^[^ ^349^ ^]^
Ag_3_PO_4_/PCN/PVA	Composite construction	Reactor: 143 mL Cat.: 0.5 g Conc.: 440 ppmv VOC: toluene Time: 80 min	Xe lamps (*λ* > 420 nm)	Techcomp 7900	Products: BZH, CO_2_ *η*(toluene): 20.79%	^[^ ^133^ ^]^
CdS/PCN	Composite construction	Reactor: 143 mL Cat.: 100 mg Conc.:10 mmol VOC: toluene Time: 180 min	Xe lamps (*λ* > 420 nm)	GCMS‐QP2010	Products: BZH, CO_2_ *η*(toluene): 83%	^[^ ^350^ ^]^
*α*‐Fe_2_O_3_/Ag/PCN	Composite construction	Reactor: 17 mL Cat.: 0.25 g Conc.:20 ppm VOC: ethylbenzene Time: 180 min	50 W white LED lamps (*λ* > 420 nm)	Spectroradiometer	Products: CO_2_ *η*(ethylbenzene): 25%	^[^ ^351^ ^]^
*α*‐Fe_2_O_3_/Cu/PCN	Composite construction	Reactor: 4.536 mL Cat.: 0.25 g Conc.:20 ppm VOC: ethylbenzene Time: unmarked	50 W white LED lamps (*λ* > 420 nm)	GC‐MS	Products: CO_2_ *η*(ethylbenzene): 37.5%	^[^ ^352^ ^]^

^a)^ The mass of the photocatalysts (Cat.)

^b)^ The concentration of Sulfur compounds (Conc.)

^c)^ Irradiation time (Time)

^d)^ The removal efficiency *η* (%) of pollutant was calculated as: *η* (%) = (1 − *C*/*C*_0_) × 100%

^e)^ Photoacoustic IR multi‐gas monitor, INNOVA Air Tech 95 Instruments Model 1412 (Innova 1412)

^f)^ High‐pressure mercury lamp (HPML)

^g)^ TU‐1901 UV–vis spectrophotometer (TU‐1901)

^h)^ POV‐18 volatile organic degradation instrument, SUNCAT, China (POV‐18)

^i)^ GAS Tiger 2000‐CH2O‐L, Shenzhen Wanandi Technology Co., Ltd. (GAS Tiger 2000‐CH2O‐L)

^j)^ Shin Kosumosu Denki Form‐tector, C‐XP‐308B (C‐XP‐308B)

^k)^ Gas chromatograph (GC)

^l)^ UV–vis spectrophotometer, UV1750, Shimadzu Corporation, Japan (UV1750)

^m)^ UV radiometer, Model UV‐A, made in the Photoelectric Instrument Factory of Beijing Normal University (Model UV‐A)

^n)^ GC/FID‐9790, Fuli, Hangzhou, China (GC/FID‐9790)

^o)^ Shimadzu GC‐8A, FID detector (GC‐8A).

#### 3.3.1 Research Progress of PCN for VOC Removal

##### Tailoring the Nanostructure

**Figure 13:**
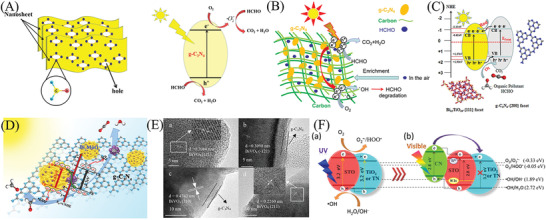
A) Schematic degradation mechanism of porous PCN nanosheet. Reproduced with permission.^[^ ^327^ ^]^ Copyright 2021, Elsevier. B) Schematic diagram of charge separation and formaldehyde photodegradation on biochar/PCN composite under visible light irradiation. Reproduced with permission.^[^ ^325^ ^]^ Copyright 2019, Elsevier. C) A schematic illustration of the facet coupled effect and electron–hole pair separation of the composite. Reproduced with permission.^[^ ^331^ ^]^ Copyright 2016, Elsevier. D) Schematic diagram of the photocatalytic oxidation of HCHO in Bi_2_MoO_6_/Bi/PCN under visible light. Reproduced with permission.^[^ ^135^ ^]^ Copyright 2020, American Chemical Society. E) TEM images of BiVO_4_/PCN samples. Reproduced with permission.^[^ ^336^ ^]^ Copyright 2017, Elsevier. F) Diagram for the band levels and the proposed electron–hole pair separation of a) STO/TN and b) STO/TN/N‐PCN heterojunctions. Reproduced with permission.^[^ ^335^ ^]^ Copyright 2017, American Chemical Society.

The porous PCN nanosheets with enhanced surface area for VOC adsorption, shortened charge transfer distance for dynamics promotion, and improved active sites for reactants in comparison with pristine PCN counterparts, have attracted attention and interest in VOC decomposition through green strategy.^[^ ^194^ ^]^ For example, Kong et al. designed and synthesized porous PCN nanosheets by treating the MA with citric acid and then thermal condensing under an N_2_ atmosphere.^[^ ^327^ ^]^ The obtained PCN nanosheets improved significantly in HCHO decomposition with LED light, which is contributed to the reduced diffusion distance of charges, improved reactive sites for HCHO (**Figure** **13**A). This work opens the avenue for PCN in the application of VOC degradation under solar irradiation.

##### Regulating Molecular Structure

The introduction of alkali metals of K into the lattice structure of PCN can rearrange the structure, lessen the lattice structure defects, and enhance basicity, which is ascribed to the formed static coulomb in the unoccupied 3d/4p orbital of K and *π*‐conjugated C–N P*_z_*.^[^ ^45^, ^49^, ^82^, ^118^, ^125^ ^]^ The increased basicity of K‐doped PCN can easily accelerate HCHO adsorption/activation/dissociation, meanwhile enhancing basicity, optical, and electrical properties, thereby showing great potential in HCHO removal.^[^ ^118^ ^]^ Therefore, the solid‐based PCN and potassium (K) were constructed and investigated in decomposing HCHO with solar light, which exhibited strengthened and reinforced performance. This investigation would shed a new way and insight for designing an advanced solid‐based PCN photocatalyst with a promoted *π*‐conjugated structure for the VOC decomposition.

In addition, the activated carbon and porous materials can physically adsorb the gaseous HCHO to reduce the concentration of the indoor/outdoor HCHO.^[^ ^325^, ^353^, ^354^, ^355^, ^356^ ^]^ However, its application is inadequate and limited because of the capability and the adsorption/desorption rate, and the lifetime of light‐excited charges. To address the above shortcomings of the activated carbon, the combination of PCN photocatalysts and activated carbon was used to improve the efficiency of the HCHO removal. The intimate interface that existed in the activated carbon and PCN endowed a prolonged lifetime and accelerated transfer kinetics of charges, thereby exhibiting outstanding HCHO decomposition with visible light.^[^ ^357^ ^]^ For instance, a novel compound (C/PCN) consisted of a biochar skeleton, and “chrysanthemum” PCN was created by the method of co‐condensed treatment.^[^ ^325^ ^]^ “Chrysanthemum” PCN increased the reaction sites and charge separation kinetics, and the biochar skeleton increased the optical absorption range and the HCHO aggregation; thus, their visible‐light removal efficiency of HCHO was 84.63%, which was higher than that of the pure PCN (Figure 13B). However, the relatively weak oxidizing ability of PCN would restrict its applications in nondegradable VOC.^[^ ^358^ ^]^ According to previous reports, the introduction of O into the PCN matrix can promote ROS generation (H_2_O_2_ production) and reduce the width of the bandgap.^[^ ^359^ ^]^ Given the above advantage, Jing et al. successfully fabricated a C, O co‐doped PCN by a facile one‐step thermal condensation.^[^ ^330^ ^]^ This photocatalyst demonstrated excellent performance in selective oxidation of toluene with visible light, which is own to the promoted ROS generation and the suppressed recombination rate of photocarriers and electronic resistance.

##### Constructing Composites

In 2019, Yao et al. used PCN nanosheets (exposing abundant hydroxyl and amino groups through the alkali treatment) to combine with the textiles through the strong hydrogen bonding interconnection, which showed efficiently decompose gaseous HCHO.^[^ ^337^ ^]^ The superior efficiency and stabilities of the PCN nanosheets could be ascribed to the ultrathin layer properties and enhanced light absorption. Rao et al. synthesized a series of Ag_3_PO_4_/PCN type II heterojunctions by introducing a certain amount of Ag_3_PO_4_ in PCN, which were used as photocatalysts in decomposing HCHO in a continuous flow.^[^ ^332^ ^]^ The formation of Ag_3_PO_4_/PCN nanocomposites increased the specific surface area for mass transfer, the separation/transfer dynamics of light‐excited electron–hole pairs, and the optical‐harvesting capability, thereby performing a high HCHO degradation ratio and reusability. The increased solar‐induced electrons on the CB were trapped by O_2_ and then reduced into ROS, whereas holes on the VB directly oxidized OH^–^ and/or water to produce ROS, and the HCHO was further oxidized to nontoxic CO_2_ and H_2_O. In 2016, Sun et al. created a series of Bi_12_TiO_20_/PCN composites via a simple method of the hydrothermal‐ultrasonic route.^[^ ^331^ ^]^ The Bi_12_TiO_20_/PCN presented higher photocatalytic activity for the HCHO degradation than that of pure PCN and Bi_12_TiO_20_ because of the active facets ({111} facets of Bi_12_TiO_20_), low index surfaces, the matched band alignment endowed promoted charge kinetics (Figure 13C).

As the above‐mentioned, the Z‐scheme heterojunction can maximize the redox ability of the semiconductor component through a zigzag charge transfer mechanism.^[^ ^49^, ^260^, ^263^ ^]^ The superfine Bi nanoparticles as a low‐cost alternative of charge transfer medium are usually used in the construction of indirect Z‐scheme nanojunctions because of the good conductivity and SPR properties.^[^ ^135^, ^360^ ^]^ In addition, Bi‐based inorganic materials show great potential in HCHO oxidization because of the band position and high‐energy orbital of Bi 6s. For instance, Wu et al. successfully constructed a solid indirect Z‐scheme nanojunction of Bi_2_MoO_6_/Bi/PCN through in situ reductions to yield Bi NPs (Figure 13D).^[^ ^135^ ^]^ This assembled nanojunction presented a commendable activity in degrading gaseous HCHO (96.15%) and robust CO_2_ selectivity (99.79%), which was primarily contributed to the small impedance endowed the decreased migration resistance of the charges, the vacancies on the surface of PCN endowed the promoted adsorption of HCHO, and the uniform distribution of Bi NPs endowed the boosted transmission of useless electrons. Tang et al. created a direct AgFeO_2_/PCN Z‐scheme composite by a simple precipitation method.^[^ ^334^ ^]^ The even loading of AgFeO_2_ particles on the surface of the layered PCN and the band alignment of Z‐scheme heterostructure endowed superior absorption in the visible‐light range, effective electron–hole separation, and maximized redox potential, thereby deeply degrading the HCHO into CO_2_ with visible light.

Except for PCN applied in the photocatalytic HCHO removal, the progress of PCN has also been made in the removal of other parts of VOC. For instance, Sun et al. synthesized a direct coral‐like Z‐scheme BiVO_4_/PCN heterojunction by a simple calcination method, which showed great improvement in the photocatalytic oxidation of toluene (Figure 13E).^[^ ^336^ ^]^ The enhancement of the efficiency was due to the kinetics of the accelerated charge favored by the Z‐scheme heterojunction, which was favorable for ROS and active site generation. Similar positive effects could be achieved by direct Z‐scheme WO_3_/PCN heterojunctions utilized in acetaldehyde decomposition with visible light. Kong et al. firstly developed a titania nanotube‐supported SrTiO_3_ heterostructure (STO/TN), followed by introducing N‐doped PCN as the sensitizer (STO/TN/N‐PCN, Figure 13F).^[^ ^335^ ^]^ This compound showed enhanced optical absorption and facilitated the charge kinetics and transfer of pollutant species, which improved visible‐light‐driven mineralization of toluene.

## 4 Conclusions and Outlook

The removal of atmospheric pollutants, including NO*_x_*, sulfur compounds, and VOC, through solar‐driven catalysis, is considered a potential solution to eliminate severe damages (human health and environment).^[^ ^9^, ^11^, ^12^, ^322^, ^323^ ^]^ Photocatalysts based on layered PCNs have demonstrated impressive performance of air pollution abatement because its layered structure favored the gas adsorption/activation/reaction, and defective structures favored the engineering and anchoring functional groups to improve charge kinetics and suitable band positions favored the ROS generation.^[^ ^45^, ^49^, ^67^, ^82^, ^172^, ^233^ ^]^ The Lewis basic sites of PCN can provide electrons to the adsorbed air pollutants and then break the bond order of N–O/C–S–C/C–H, thereby facilitating the conversion of air pollutants.^[^ ^12^, ^17^, ^103^, ^104^ ^]^ In addition, the visible‐light‐response PCN provides great potential and value for commercialization, which is different from the traditional TiO_2_ photocatalysts.^[^ ^45^, ^49^, ^82^ ^]^ This review explains the strategies to optimize and improve the efficiency of PCN‐based photocatalytic denitrification, desulfurization, and VOC removal through nanostructure design/electronic structure engineering/heterostructure manufacturing. Firstly, nanostructured PCN‐based composites prepared by exfoliation/templating/solvothermal technologies, and other methods show an excellent catalytic degradation efficiency of air pollutants compared with the pristine PCN counterpart with visible light. The morphological regulation, vacancy/defect engineering, and catalytic immobilization can modulate the surface/internal structure and photo/electrochemical characters to reduce the charges transfer distance, promote mass diffusion, enhance its reactants adsorption/activation, and products desorption, separate the powdery photocatalysts, and decrease photocatalytic loss. Secondly, reforming the electronic structure of PCN‐based photocatalysts through atomic and molecular doping can introduce extra impurities, adjust the redox potentials of charge carriers and optical absorption capability, and extend the delocalization of the *π* electrons, thereby promoting the efficiency of multiphase catalysis for the elimination of air pollutants. Finally, the heterostructure photocatalysts by combining PCN and other functional materials based on matching band and band alignment are found to be efficacious, which can improve the kinetics of light‐induced charge carriers and exhibit outstanding heterogeneous catalysis for the decomposition of air pollutants. In addition, the above‐mentioned solutions can also improve the air molecules activation and the ROS generation, further advancing the denitrification, desulfurization, and VOC removal. Thus, the photocatalytic efficiency of PCNs would be significantly enhanced more rationally with the structure design of PCN through different strategies. The potential results discussed and analyzed in this review can inspire scientists to develop more effective PCN‐based photocatalytic composites to reduce and degrade the air pollutants concentration.

Although many advances have been achieved, the PCN‐based materials still cannot be commercially applied because of the issues of uncertain generation/conversion routes of by‐products, low performance with red‐light, nonuniform universal evaluation standards, limited/complicated simulation tools/technologies, inefficient durability, and reusability of photocatalysts, and photocatalytic regeneration. Therefore, establishing a complete evaluation system and conducting a basic exploration and research work is necessary to resolve these technical problems and challenges and accelerate the progress of commercialization. In our opinion, priority research soon should concentrate on the development of PCN‐based photocatalysts with the following demonstrated properties. 1) Lack of universal evaluation standards: Determining the actual efficiency of the photocatalysts is difficult to evaluate because of the diverse test systems. 2) Unsatisfactory and moderate photocatalytic activity: Although various PCN‐based photocatalysts have been reported and investigated in removing the air pollution, the actual efficiency is still too unsatisfactory and low to realize the practical needs. 3) Lack of infrared region utilization: The infrared region utilization of PCN should be developed to achieve greater improvement in solar energy efficiency. 4) Poor product selectivity: Considering a multilevel reaction of removing air pollutants takes place on PCN photocatalysts, a variety of toxic and harmful by‐products appear during the reaction. Therefore, reducing the production of toxic by‐products and deep oxidation to remove air pollutants is an essential step. 5) Poor and inadequate operational stability: Operational stability is one of the first prerequisites for the industrial application of catalysts. However, the current research on the operational stability of PCN photocatalysts still has many limitations, and the research is not yet in‐depth. 6) Lack of catalytic immobilization carrier and technology: Recycling and reusing PCNs during photocatalytic air abatement are difficult. Since the powdered photocatalyst is easily blown away under the continuous gas during the practical application, it causes loss of catalyst; and it also requires a time‐consuming and complicated filtration process during the recycling process, which increases the process cost. 7) Lack of in‐depth mechanism research: To substantially improve the reaction efficiency of photocatalytic removal of air pollutants, the reaction process needs to be identified through various advanced in situ detection technologies and theoretical calculations. More in‐depth researches are needed to break the current bottleneck in the next decades. Moreover, to further realize the large‐scale application of PCN‐based photocatalysts, its preparation process, cost and output are also major factors that still need to be considered. Thus, more efforts are still needed to devote to designing and producing low‐cost PCN‐based nano‐photocatalysts with superior activity, selectivity, and reusability and achieve the maximization of the above properties.

A few further optimizations can be considered with regard to PCNs as follows. 1) Establishment of a standard test and judge system: The standard and the publicly recognized testing system can be built by internationally recognized experts to further improve the catalytic system and efficiency. 2) Crystal structure modulation: The crystallinity and defects modification of PCN can be devised to increase the kinetics of the charge and provide enhanced adsorption sites for reactants.^[^ ^85^, ^100^ ^]^ 3) Morphological modification: The PCN aerogels, mesoporous PCN, and ultrathin/single‐layer PCN nanosheets can be designed to promote the mass transfer and shorten the transmission distance of the photogenerated charges.^[^ ^97^, ^99^, ^100^, ^142^, ^143^, ^145^, ^361^, ^362^, ^363^ ^]^ 4) Immobilization base and process regulation: The increased stability and extended life of PCNs can be achieved by selecting suitable immobilization bases and improving the fixing process. 5) Atomic utilization promotion: The single‐atom‐based PCN photocatalytic systems can be designed and constructed to maximize atomic utilization.^[^ ^364^, ^365^, ^366^ ^]^ In addition to finding and designing suitable component materials to combine with PCN, its synthesis techniques and processes are also indispensable for the photocatalytic performance improvement and commercial development of its surface modification. Through the development of nanotechnology, solar‐driven engineering, and theoretical/model research fields, its photocatalytic performance can be significantly improved and large‐scale industrialization and commercial application of these materials can be realized. Given such optimizations, improving the efficiency of air purification based on PCN‐based photocatalysts and other solar‐related applications would be recommended.

## Conflict of Interest

The authors declare no conflict of interest.
